# A single-molecule localization microscopy method for tissues reveals nonrandom nuclear pore distribution in *Drosophila*

**DOI:** 10.1242/jcs.259570

**Published:** 2021-12-16

**Authors:** Jinmei Cheng, Edward S. Allgeyer, Jennifer H. Richens, Edo Dzafic, Amandine Palandri, Bohdan Lewków, George Sirinakis, Daniel St Johnston

**Affiliations:** 1 The Gurdon Institute and Department of Genetics, University of Cambridge, Tennis Court Road, Cambridge CB2 1QN, UK; 2 Institute of Reproductive Medicine, School of Medicine, Nantong University, Nantong 226001, China

**Keywords:** Super-resolution microscopy, DNA-PAINT, Nuclear pore complex, *Drosophila*, Lamin C

## Abstract

Single-molecule localization microscopy (SMLM) can provide nanoscale resolution in thin samples but has rarely been applied to tissues because of high background from out-of-focus emitters and optical aberrations. Here, we describe a line scanning microscope that provides optical sectioning for SMLM in tissues. Imaging endogenously-tagged nucleoporins and F-actin on this system using DNA- and peptide-point accumulation for imaging in nanoscale topography (PAINT) routinely gives 30 nm resolution or better at depths greater than 20 µm. This revealed that the nuclear pores are nonrandomly distributed in most *Drosophila* tissues, in contrast to what is seen in cultured cells. Lamin Dm_0_ shows a complementary localization to the nuclear pores, suggesting that it corrals the pores. Furthermore, ectopic expression of the tissue-specific Lamin C causes the nuclear pores to distribute more randomly, whereas *lamin C* mutants enhance nuclear pore clustering, particularly in muscle nuclei. Given that nucleoporins interact with specific chromatin domains, nuclear pore clustering could regulate local chromatin organization and contribute to the disease phenotypes caused by human lamin A/C laminopathies.

## Abstract (precis)

**Summary:** Description of a simple line scanning microscope for super-resolution imaging of tissues using DNA-PAINT. This reveals a non-random distribution of nuclear pores in most *Drosophila* cell-types.

## INTRODUCTION

The development of super-resolution microscopy techniques that break the diffraction limit has enabled the visualization of subcellular structures with unprecedented resolution (Galbraith and Galbraith, 2011; Vangindertael et al., 2018). Some of the highest resolutions are provided by a range of single-molecule localization microscopy (SMLM) approaches that separate the fluorescence produced by closely spaced fluorophores in time, so that each camera frame captures the image of only a few sparsely distributed, active emitters (Betzig et al., 2006; Hess et al., 2006; Rust et al., 2006). The image of each emitter is fitted with a model function, typically a two-dimensional (2D) Gaussian, to localize the emitter position. Repetitively localizing thousands or millions of single-emitter positions over many camera frames results in a reconstructed final image with a resolution below the diffraction limit, typically on the order of 20 to 30 nm.

The localization precision, and subsequently the resolution, achieved by SMLM depends on the number of photons from each blink and the background, primarily from out-of-focus emitters in the case of DNA-PAINT. However, this has largely constrained SMLM to thin samples close to the coverslip, where the background is low. The resolution can be further enhanced by reducing the background with illumination configurations that restrict the excitation of the fluorophores to a narrow axial region, such as total internal reflection fluorescence (TIRF), highly inclined and laminated optical sheet illumination (HILO) or selective plane illumination (SPIM) (Chen et al., 2014; Tokunaga et al., 2008; van den Dries et al., 2013).

SMLM imaging of thick tissues is more challenging because of the high levels of background fluorescence from out-of-focus emitters and tissue autofluorescence, as well as the reduced number of photons collected from each emission due to aberrations or scattering. Chemical fluorophores have higher stability and quantum yields than fluorescent proteins, making SMLM techniques that are based on fluorescent dyes better suited for tissue samples. In the most common SMLM technique, stochastic optical reconstruction microscopy (STORM), specific ‘blinking’ dyes are conjugated to the protein of interest, switched into a dark state and then imaged as they randomly reactivate in a special ‘blinking buffer’ (Huang et al., 2009; Rust et al., 2006). The range of dyes that can be induced to blink is limited, however, and each dye has optimal buffer conditions and blinking characteristics, which complicates multicolour imaging. Photobleaching can also reduce image quality as fluorophores that bleach before they are imaged are not detected (Diekmann et al., 2020). These problems are largely avoided by an alternative labelling approach called DNA-based point accumulation for imaging in nanoscale topography (DNA-PAINT), in which the protein of interest is labelled with a docking oligonucleotide (the docking strand) and then imaged using the complementary oligonucleotide (the imager strand) fused to a fluorescent probe (Jungmann et al., 2010). The imager strand diffuses rapidly in solution and appears as background, but remains stationary on hybridizing to the docking strand, producing a ‘blink’ of light that can then be localized. Because the fluorophore is not directly conjugated to the protein of interest and there is a large excess of imager strand in solution, the sample does not suffer from photobleaching. One can therefore image for longer and collect many ‘blinks’ from the same labelled protein. DNA-PAINT also removes the need to use fluorophores that ‘blink’ in specialized buffers, and one can therefore more flexibly choose pairs of spectrally separated fluorophores with high photon yields, improving two-colour imaging. On the other hand, DNA-PAINT has higher background due to fluorescence from freely diffusing imager strands.

One imaging modality that improves imaging performance in thick samples is confocal microscopy, which relies on a mechanical pinhole to reject out-of-focus light. Confocal microscopy is most often realized in a point scanning geometry, which is unsuitable for SMLM imaging because it is intrinsically slow. However, camera-based variants such as spinning disc or line scanning confocal microscopes have recently been employed in SMLM to image the entire thickness of cells and, in some cases, tissue (Park et al., 2018; Schueder et al., 2017). However, even with these developments, SMLM is generally limited to cultured cell applications, as the localization precision deteriorates with imaging depth in high-background environments.

Although line scanning is usually implemented using a slit to reject out-of-focus light, several groups have instead used the rolling shutter capabilities of commercially available scientific complementary metal–oxide–semiconductor (sCMOS) camera sensors for confocal or light-sheet imaging (Baumgart and Kubitscheck, 2012; Kumar et al., 2016; Mei et al., 2012). Here, we adapt the rolling shutter approach to perform DNA-PAINT imaging in tissue samples with high background. Compared to previous line scanning implementations, our approach eliminates the use of descan optics and offers a simpler and more efficient design (Lee et al., 2012; Tang and Han, 2018). We present a pipeline for performing multi-colour SMLM on endogenous proteins in tissue samples, with optimized protocols for labelling SNAP- and Halo-tagged proteins with docking strand oligonucleotides and an exchangeable single-molecule localization (IRIS) probe for visualizing F-actin as a landmark. We tested the line scanning system using endogenously-tagged nucleoporins and discovered that the nuclear pores are highly clustered in many *Drosophila* tissues, providing the first evidence for the non-random distribution of nuclear pores in wild-type cells.

## RESULTS

### Design of a microscope for PAINT imaging in tissues

**Fig. 1.**
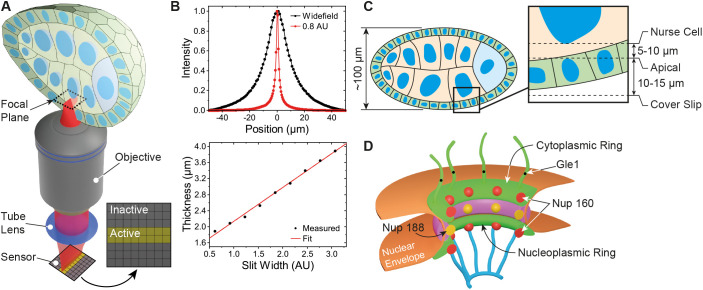
**Microscope and sample geometry overview.** (A) Excitation light is focused into a line at the sample plane inside the egg chamber. Fluorescence is collected by the objective lens and replayed to an sCMOS camera chip where active and inactive pixel rows form an electronic slit for optical sectioning. (B) Optical thickness comparison between wide-field (normal) and slit scanning modes. An optical section of ∼1.5 µm is formed with the electronic slit set to 0.8 airy units (AU). (C) Detailed view of a typical image focal plane. The apical membrane resides 10–15 µm inside the egg chamber, while the nurse cell nucleus is an additional 5–10 µm further depending on egg chamber stage. (D) Schematic of a nuclear pore complex. Nup160 resides in the outer cytoplasmic and nucleoplasmic rings, Nup188 lies in the inner rings and Gle1 is a component of the cytoplasmic filaments.

To perform optical sectioning of thick samples, we designed and built an inexpensive line scanning confocal system using standard optical components (Fig. 1A; Fig. S1A,B). Briefly, an excitation laser is focused into a line with diffraction limited width at the sample and scanned across the field of view (FOV) with a galvanometer mirror. Fluorescence is collected with the same objective and relayed to an sCMOS camera. In contrast to most previous implementations of confocal line scanning that employed a mechanical slit, we exploited the flexible architecture of the sCMOS camera and used rows of active and inactive pixels to form an ‘electronic’ slit (Kumar et al., 2016; Mei et al., 2012) that rejects out-of-focus light (Baumgart and Kubitscheck, 2012; Tang and Han, 2018). By adjusting the time delay between neighbouring rows, one can set the speed with which the active pixel rows move across the camera sensor and synchronize it with the scanning excitation line to form an image. The width of the electronic slit can be controlled by the software and has the same effect as opening or closing a confocal pinhole to adjust the optical section thickness (Fig. 1B). In this way, pixel integration time, slit width and scanning speed can be optimized to maximize signal collection and minimize out-of-focus background. The use of an ‘electronic’ slit minimizes mechanical parts and allows for a simple optical setup with tuneable optical sectioning superior to wide-field illumination (Fig. 1B).

### Testing microscope performance by imaging nuclear pore complex in tissues

To examine the performance of the microscope, we imaged *Drosophila* egg chambers (Fig. 1C), as these are amenable to genetic manipulation and large enough to test the optical sectioning capabilities of the system. The nuclear pore complex (NPC) has been proposed as a reference structure for super-resolution microscopy, since it is abundant in the nuclear envelopes of all cells and has a known structure and stoichiometry, with a characteristic 8-fold symmetry (Hampoelz et al., 2019; Thevathasan et al., 2019). To image endogenous proteins using DNA-PAINT, we used CRISPR-mediated homologous recombination to introduce the self-labelling SNAP and Halo tags into three nucleoporins that localize to different parts of the NPC – Nup160, which is present in two copies in each of the eight spokes of the cytoplasmic and nuclear outer rings, Nup188, which is found in 32 copies in the inner ring, and Gle1, a component of the cytoplasmic filaments that extend from near the centre of the NPC (Fig. 1D) (Gautier et al., 2008; Hampoelz et al., 2019; Los et al., 2008).

To perform DNA-PAINT imaging of nuclear pores, we optimized conditions for conjugating docking strands to the tagged nucleoporins. Docking strands can be efficiently conjugated to SNAP and Halo-tagged proteins by adding O6-benzylguanine or the chloroalkane Halo tag ligand to the 5′ end of the oligonucleotide (Fig. S1C), as previously reported (Schlichthaerle et al., 2019). Promega also kindly provided us with a compound, PBI-300-43, that can be attached to a 12-carbon linker on the 5′ end of the oligonucleotide to produce a modified docking strand that is a better substrate for the Halo tag in some *in vitro* assays (Fig. S1D,E). Both chloroalkane-modified oligonucleotides conjugated equally efficiently to the Halo-tagged proteins in fixed and permeabilized *Drosophila* egg chambers. We measured the effective labelling efficiency (EL) for Halo by imaging Nup160 and Gle1 in flies that were homozygous for Nup160–SNAP and heterozygous or homozygous for Gle1–Halo, and scoring the proportion of Nup160-marked nuclear pores that contained Gle1–Halo signal in the centre. There are eight copies of Gle1 per nuclear pore and the probability of any Gle1 molecule being detected in Gle1 heterozygotes is half the effective labelling efficiency because only one of the two copies are tagged. The probability of a nuclear pore containing no detected Gle1 molecules is therefore 
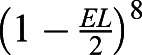
 for Gle1 heterozygotes and (1−*EL*)^8^ for homozygotes allowing us to calculate EL from the frequency of nuclear pores with no Gle1 signal. These measurements gave effective labelling efficiencies of 20–40%, with fresh docking strands giving higher labelling (Table S1). This is comparable to the labelling efficiencies obtained when conjugating fluorescent dyes to Halo-tagged proteins (Thevathasan et al., 2019).

**Fig. 2.**
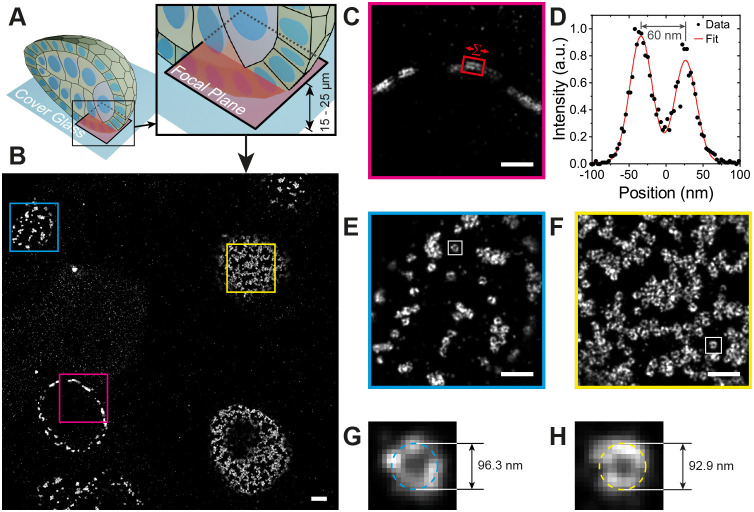
**Nup160 DNA-PAINT egg chamber imaging.** (A) An illustration of the position of the focal plane imaged in (B), which is 15 to 25 µm above the coverslip and inside the egg chamber. (B) DNA-PAINT image showing nurse cell and follicle cell nuclei. (C,D) A magnified view of the area outlined by the red box in B showing a cross-sectional view of NPCs in the nuclear membrane (C). The intensity distribution between the outer and inner rings of the NPCs in region in C is shown in D, with the distance measured as 60 nm. (E,F) Detailed views of NPCs in the follicle cell nuclear membrane (E) and the nurse cell nuclear membrane (F), showing distinct ring structures with hollow centres. (G,H) Detailed views of NPCs from E and F fitted with a circle and diameters of 96.3 and 92.9 respectively. Data shown are representative of 27 images. Scale bars: 1 µm (A); 500 nm (C,E,F).

We used this method to perform DNA-PAINT of the nuclear pores in the follicle cells and germ cells (Fig. 2A,B) of *Drosophila* egg chambers homozygous for Nup160–Halo. Since DNA-PAINT is resistant to photobleaching, we could routinely collect 50,000 to 100,000 camera frames with only a 20–40% reduction in the number of blinks per frame by the end, presumably due to photodamage to the docking strands when bound imager strands are bleached (Fig. S2A) (Blumhardt et al., 2018). We obtained between 600 and 2600 photons per blink (Fig. S2B), depending on the imaging conditions, to give a localization precision between 8 and 14 nm (Fig. S2C,D), resulting in a maximum achievable resolution of 19 to 33 nm. This resolution allowed us to measure the distance between the nuclear and cytoplasmic rings of the NPC (Fig. 2C,D) in our images, giving an average of 57.7±4.4 nm (mean±s.d., *n*=99, Fig. S3A), which corresponds well with the distance measured from electron microscopy (EM) data (von Appen and Beck, 2016). The mean NPC diameter was also measured by performing a circular fit on individual NPCs (Fig. 2E–H) and found to be 103.4±6.5 nm (mean±s.d., *n*=226, Fig. S3B), in good agreement with the expected value from EM on *Drosophila* NPCs and super-resolution studies of NPCs in other organisms (Kiseleva et al., 2001; Thevathasan et al., 2019). The nurse cell nuclei lie ∼10 µm above the follicle cell epithelium, which is at least 5 µm thick. Thus, the imaging plane in Fig. 2 is ≥15 µm into the sample, demonstrating that our line scanning system can collect high quality super-resolution images deep inside tissues.

### Imaging F-actin using Lifeact peptide-PAINT

**Fig. 3.**
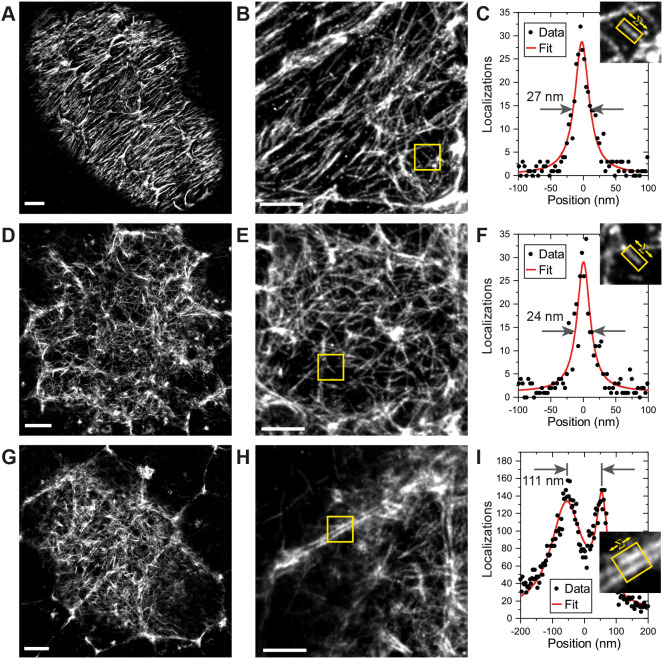
**Peptide-PAINT imaging in *Drosophila* egg chambers.** (A) Basal F-actin in a stage 5 *Drosophila* egg chamber. (B) Detailed view from A showing that smaller filaments are well-resolved. (C) A single filament from the boxed region in B is shown on the upper right. The graph shows the distribution of localizations perpendicular to the straight filament boxed in yellow after summing along its long axis (see Materials and Methods for details). The filament diameter was measured as 27 nm FWHM by fitting with a Gaussian. (D) Apical F-actin in a stage 5 *Drosophila* egg chamber. (E) Detailed view from B showing small filaments are visible. (F) The box region in E is shown at the upper right. The graph shows the distribution of localizations perpendicular to the straight filament boxed in yellow after summing along its long axis. The filament diameter was measured as 24 nm FWHM by fitting with a Gaussian. (G) Apical F-actin in a stage 8 *Drosophila* egg chamber. (H) Detailed view from G showing the clear separation between the cortical F-actin on adjacent cell membranes. (I) The boxed region in H is shown on the upper right. The graph shows the distribution of blinks perpendicular to the regions of the cell cortices boxed in yellow after summing the localizations along each cell cortex. Fitting with two Gaussians gives the distance between the membranes as 111 nm. Data shown are representative of 8 images. Scale bars: 2 µm s (A,D,G); 1 µm (B,E,H).

When imaging less well-characterized proteins that do not localize to known structures, it is important to have landmarks to indicate where in the cell the protein is localized. The actin cytoskeleton represents an excellent landmark, as it outlines the cell cortex and forms specific structures in different regions of the cell. We therefore adapted the IRIS method for labelling F-actin using the Lifeact peptide, which binds to F-actin with high specificity, but low affinity (Kiuchi et al., 2015). To increase the stability of the probe, we added an extra cysteine residue to the N-terminus of the peptide and conjugated the organic fluorescent dye, Cy3B, to this residue through a stable thioether bond using Cy3B-maleimide (Nanda and Lorsch, 2014). In this peptide-PAINT approach, transient binding of the Cy3B-Lifeact peptide to F-actin results in blinks that are imaged in the same way as DNA-PAINT. We imaged the actin organization in both the basal and apical side of follicles cells in stage 5 *Drosophila* egg chambers (Fig. 3). Stage 5 egg chambers rotate around their anterior-posterior axes due the coordinated circular migration of the follicle cells, driven by planar polarized lamellipodia and stress fibres on their basal surfaces (Cetera et al., 2014; Haigo and Bilder, 2011). Super-resolution Lifeact imaging of the basal surface showed the planar polarized stress fibres with thinner actin filaments branching out from them, while the high density of actin in the lamellipodia appeared as bright regions in the image (Fig. 3A,B). The thinner filaments (Fig. 3C) have an apparent diameter of 30.4±8.42 nm (mean±s.d., *n*=11; Fig. S3C) with the smallest features being below 20 nm, and offer an indication of the resolving power of the microscope. By contrast, the actin on the apical surface of stage 5 follicle cells forms a branched network, with densely labelled nodes that may correspond to Myosin puncta (Balaji et al., 2019) (Fig. 3D,E,F). The apparent diameter of the filaments was 30.2±4.48 nm (mean±s.d., *n*=10; Fig. S3D), with the smallest features being below 20 nm, which was comparable to that on the basal side, demonstrating that the performance of the microscope is not significantly affected over a depth of ∼10 µm. This suggests an image resolution at or better than 40 nm under our experimental conditions (sample drift, labelling density, and photon and background levels). By stage 8 of oogenesis, the apical actin network has become denser, with higher accumulations of actin at the tri-cellular junctions (Fig. 3G,H). The higher resolution also allowed us to resolve the actin cortices in adjacent cells, which are separated by 111.0±15.7 nm (mean±s.d., *n*=11; Fig. 3I; Fig. S3E).

### Two-colour imaging using DNA- and peptide-PAINT

**Fig. 4.**
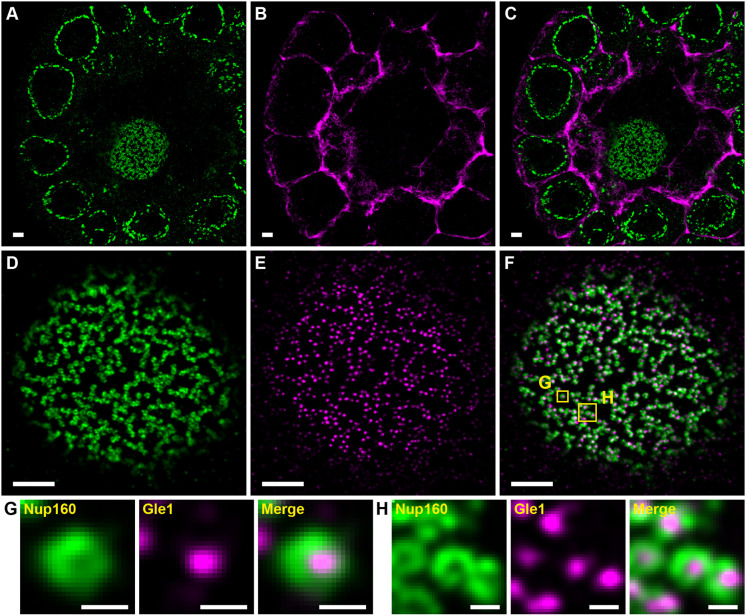
**Combined DNA- and Peptide-PAINT two-colour imaging.** (A–C) Nup160 visualized using DNA-PAINT (A) and imaged simultaneously with F-actin (B) visualized with Peptide-PAINT. (C) Merge of A and B. (D–F) Nup160 (D) and Gle1 (E) imaged simultaneously with DNA-PAINT. The merged image is shown in F. (G) An enlarged view of the left-hand boxed region (labelled G) in F showing the Nup160 signal, the Gle1 signal and the merged image. (H) A similar enlarged view of the right-hand boxed region (labelled H) in F. Gle1 appears inside the Nup160 rings as expected. Data shown are representative of 5 images for Nup160/F-actin and 77 images for Nup160/Gle1. Scale bars: 1 µm (A–F); 100 nm (G,H).

Imaging of multiple molecules with DNA-PAINT has been demonstrated using Exchange-PAINT in which different molecules are imaged sequentially after changing the imaging oligonucleotides (Jungmann et al., 2014). However, imaging two molecules takes twice as long as imaging one and exchanging imager oligonucleotides increases the possibility of sample drift. In this work, typical image acquisition times range between 3 and 5 h with significant sample drift, making Exchange-PAINT impractical. We therefore tested whether one can perform DNA-PAINT and peptide-PAINT simultaneously by imaging F-actin and Nup160 in stage 3 egg chambers (Fig. 4A–C). The Lifeact signal highlights the actin-rich cortex in every cell, revealing the arrangement of the follicle cells around the nurse cells, which lie internally (Fig. 4C). Thus, Lifeact-PAINT provides a useful reference system for showing the cell boundaries and locating the imaging plane within the sample, in this case more than 20 µm above the coverslip. We also tested simultaneous two-colour DNA-PAINT by imaging egg chambers from Nup160–SNAP Gle1–Halo homozygous females using two different docking strand oligonucleotides and complementary imager strands labelled with Cy3B and Atto643 (Fig. 4D–F). This reveals the localization of Gle1 in the centre of each NPC surrounded by rings of Nup160 (Fig. 4G,H).

### Nuclear pores are clustered in *Drosophila* egg chamber follicular and nurse cells

**Fig. 5.**
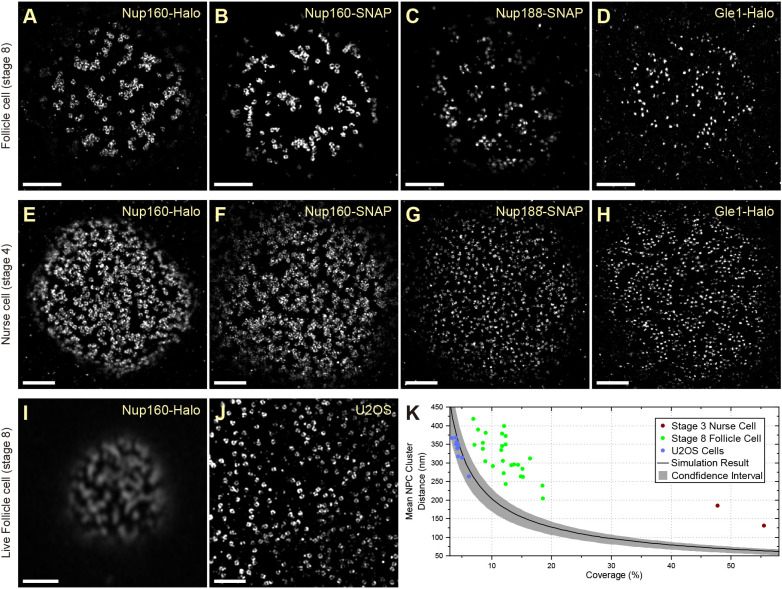
**Super-resolution imaging in tissue reveals a nonrandom distribution of NPCs.** (A–D) DNA-PAINT images of Nup160–Halo (A), Nup160–SNAP (B), Nup188–SNAP (C) and Gle1–Halo (D) in NPC in stage 8 follicle cell nuclei. The NPC appear clustered, regardless of the protein/tag combination used, showing that the organization is not caused by the tag. (E–H) DNA-PAINT images of Nup160–Halo (E), Nup160–SNAP (F), Nup188-SNAP (G) and Gle1–Halo (H) in NPCs of stage 4 nurse cell nuclei. A similar NPC clustering is seen in nurse cell nuclei, suggesting that this organization is not specific to the follicular epithelium. (I) Live imaging of JF646-labelled Nup160–Halo in a stage 8 follicle cell nucleus using a Zeiss Airyscan microscope. The clustering is present *in vivo*, indicating that it is not caused by fixation. (J) DNA-PAINT image of Nup96–Halo in a cultured U2OS cell nucleus. The NPCs appear uniformly distributed without any underlying organization. (K) A graph showing the mean NPC cluster distance as a function of the proportion of the nuclear envelope covered by NPCs (coverage). The black line shows the mean distances obtained from simulations of random NPC distributions, with 95% confidence intervals shown in grey. U2OS cells show a random distribution of NPCs, whereas the mean distances between NPC clusters are significantly larger in follicle cell and nurse cell nuclei, indicating that the NPCs are non-randomly distributed. See Materials and Methods for analysis details. Scale bars: 1 µm (A–H,J); 5 µm (I).

A striking feature of the super-resolved images of nuclear pores (NPCs) in the follicle cells is their organization into inter-linked chains separated by nuclear pore-free areas, a seemingly non-random distribution (Fig. 2B,E,F). This is surprising, since the nuclear pores are thought to be evenly distributed around the nuclear envelope and previous super-resolution images have revealed an even distribution of NPC in tissue culture cells (Daigle et al., 2001; Dultz and Ellenberg, 2010; Rabut et al., 2004; Schlichthaerle et al., 2019; Thevathasan et al., 2019). One possibility is that this apparent clustering is a consequence of the fusion of the Halo tag to Nup160. To test this, we compared the distributions of Nup160–Halo, Nup160–SNAP, Nup188–SNAP and Gle1–Halo in the follicle cells at stage 8 of oogenesis and the nurse cells at stage 4 (Fig. 5). Nup160–Halo and Nup160–SNAP show indistinguishable nuclear pore distributions in both the follicle cells and the nurse cells, which have a higher nuclear pore density, but still show a pronounced tendency to cluster (Fig. 5A,B,E,F). Furthermore, Nup188–SNAP and Gle1–Halo show very similar distributions of NPCs in both tissues, although they lie nearer to the centre of the nuclear pores than Nup160 (Fig. 5C,D,G,H). Thus, the non-random distribution of the nuclear pores is not caused by the Halo tag or fusions to Nup160. Finally, we tested whether the clustering is a fixation artefact by imaging Nup160–Halo in living egg chambers after labelling the Halo tag with the cell permeable dye JF646 (Grimm et al., 2015) (Fig. 5I). Despite the lower resolution of the Zeiss Airyscan microscope (∼120 nm), the ribbons of clustered nuclear pores are clearly visible, demonstrating that this is the organization of the nuclear pores *in vivo*.

To quantify the spatial distribution of nuclear pores, we developed an automated pipeline that measures the distances between nuclear pore clusters. As the NPCs become more clustered, the mean distance between clusters increases and this is therefore a proxy measure for clustering. The distances between nuclear pore clusters are also influenced by the density of nuclear pores in the nuclear membrane (coverage) and their labelling efficiency. To estimate how these two parameters affect the nuclear pore clustering, we performed simulations in which the nuclear pores are distributed randomly without overlapping, but with different densities and labelling efficiencies (Fig. S4A–C). Using the data from these simulations, we established a baseline for random nuclear pore distribution along with confidence intervals that can be used as a reference over a wide range of nuclear pore densities and labelling efficiencies (Fig. S4D). To test this analysis pipeline, we imaged Nup96–Halo in U2OS cells, where the nuclear pores are evenly distributed (Thevathasan et al., 2019) (Fig. 5J). Almost all the measured mean NPC cluster distances in U2OS nuclei fall within the confidence intervals of the simulations (Fig. 5K), demonstrating that the NPC distribution is close to random. By contrast, the mean NPC cluster distances in the follicle cells and nurse cells are significantly larger (Fig. 5K), indicating that the nuclear pores are significantly more clustered than one would expect by chance.

### Nuclear pores are clustered in different *Drosophila* tissues

**Fig. 6.**
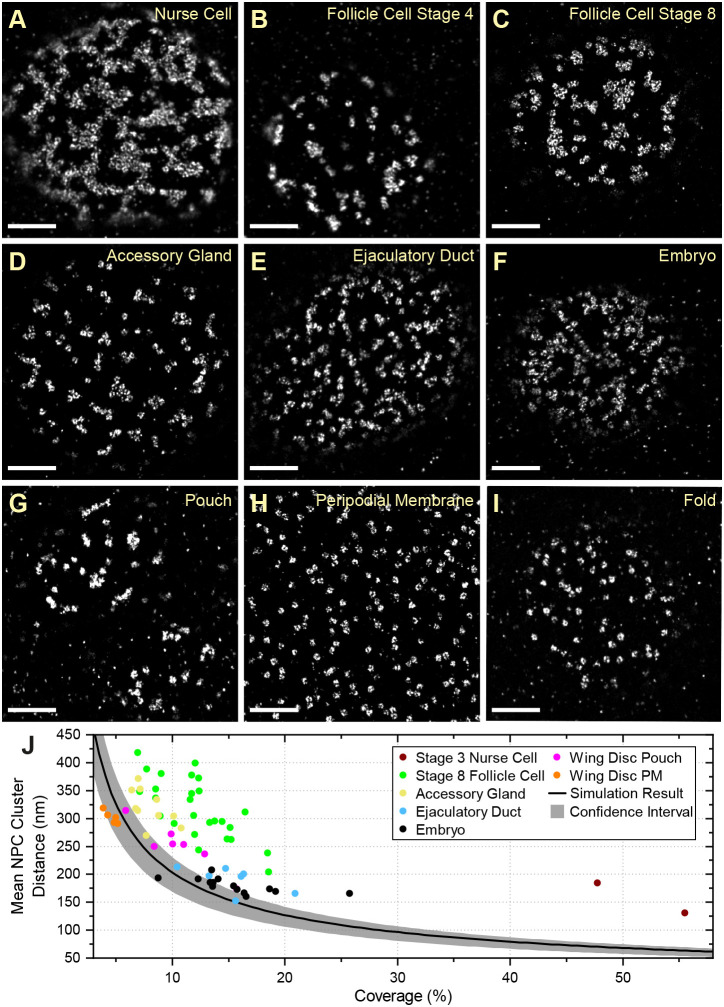
**NPC organization in different *Drosophila* tissues.** NPC clustering is observed in nurse cells (A) and follicle cells at multiple stages (B–C). Similar NPC clustering is observed in the accessory gland (D), the ejaculatory duct (E), the syncytial blastoderm embryo (F) and the pouch region of the wing imaginal disc (G). This organization is not observed in the peripodial membrane of the wing disc (H) or the wing disc fold (I). (J) A graph showing the mean NPC cluster distance as a function of the proportion of the nuclear envelope covered by NPCs for each tissue. The peripodial membrane (wing disc PM) has a similar mean NPC cluster distance to the simulations of random NPC distributions, whereas all other tissues show significantly greater cluster distances, indicating a clustered distribution. The wing disc fold was not included in this analysis because the sample size is too small. Scale bars: 1 µm.

To determine whether nuclear pore clustering is a common feature of *Drosophila* cells, we examined the localization of Nup160–Halo in other tissues. The NPCs in the nuclei of the testis accessory gland and ejaculatory duct of adult males show a similar degree of clustering to the follicle cells (Fig. 6A–E). We also examined the arrangement of nuclear pores in the nuclei of the syncytial blastoderm embryo (Fig. 6F) and observed that most nuclei show some clustering, falling outside the 95% confidence limits for a random distribution (Fig. 6J). This case also appears to be the case in nuclei that have just exited mitosis, indicating that clustering is already present when the nuclear envelope reforms after mitosis (Fig. S5). In the third-instar imaginal wing disc, the nuclear pores appear clustered in the nuclei of the pouch region (Fig. 6G), whereas the peripodial membrane and the fold show no apparent clustering (Fig. 6H,I). Thus, the nuclear pores are clustered at all stages of development and in most tissues analysed, regardless of whether they are dividing progenitor cells or terminally differentiated post-mitotic cells.

Given that the nuclear pores span the nuclear envelope, their organization could be dictated by either cytoplasmic or nuclear signals. Nuclear pore clustering could be mediated by the cytoskeleton, which interacts with and exerts force on the nuclear envelope both by the direct binding of motors to the nuclear pores and through the LINC complex, which spans the nuclear envelope and can bind to F-actin and microtubules (Bolhy et al., 2011; Guo and Yixian, 2015; Jahed et al., 2016; Soheilypour et al., 2016; Splinter et al., 2010). We therefore tested whether either microtubules or F-actin are required for the distribution of nuclear pores by treating egg chambers with the microtubule-depolymerizing drug colcemid or the F-actin-depolymerizing drug Latrunculin A. Despite the loss of almost all microtubules or F-actin in treated egg chambers, neither drug had any effect on the distribution of nuclear pores in either the follicle cells or the nurse cells (Fig. S6).

### Lamin C is connected to NPC clustering and Lamin Dm_0_

**Fig. 7.**
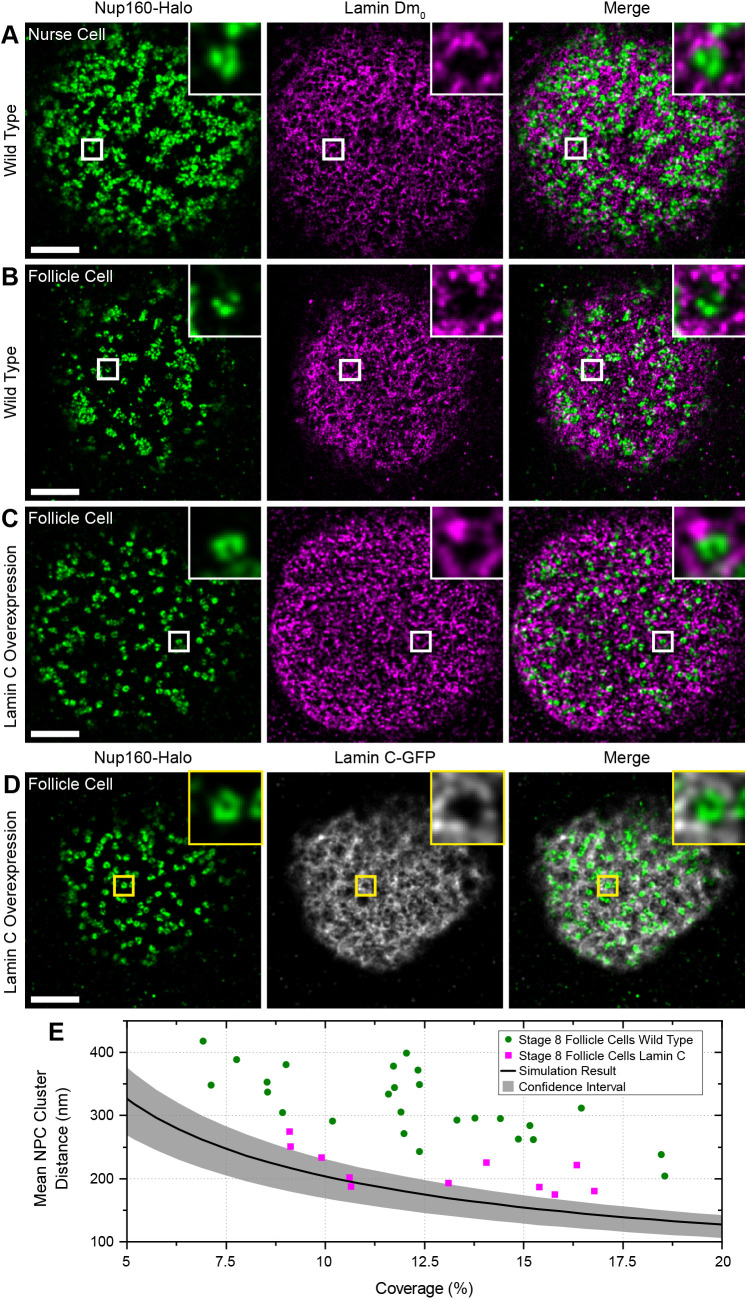
**Lamin C inhibits NPC clustering.** (A) DNA-PAINT image of Nup160–Halo (green) and Lamin Dm_0_ (magenta) in a wild-type nurse cell. The NPC clusters mainly fall in the gaps in the Lamin matrix. (B) DNA-PAINT image of Nup160–Halo (green) and Lamin Dm_0_ (magenta) in a wild-type follicle cell nucleus. Again, the NPC clusters anti-correlated with Lamin Dm_0_ (column 2). (C) DNA–PAINT image of Nup160–Halo (green) and Lamin Dm_0_ (magenta) in a follicle cell nucleus over-expressing GFP- Lamin C. The NPCs are less clustered, and Lamin Dm_0_ appears to form shorter and more globular clusters. (D) DNA-PAINT image of Nup160–Halo (green) and GFP–Lamin C (greyscale) in a follicle cell nucleus over-expressing GFP–Lamin C. (E) A graph showing the mean NPC cluster distance as a function of the proportion of the nuclear envelope covered by NPCs for wild-type and Lamin C-expressing follicle cell nuclei. The Lamin C-expressing nuclei show a more random distribution of NPC. Scale bars: 1 µm.

The nuclear pores are static in the nuclear envelope because they are anchored to the nuclear lamina through multiple interactions between filaments formed by Lamin proteins and specific nucleoporins, making the Lamins good candidates for factors that position the nuclear pores (Daigle et al., 2001; Lussi et al., 2011; Smythe et al., 2000; Walther et al., 2001). The follicle cells and nurse cells express only one Lamin at detectable levels, the B-type Lamin called Lamin Dm_0_ (Melcer et al., 2007). We therefore performed two-colour DNA-PAINT imaging of Lamin Dm_0_ and Nup160–Halo using an anti-Lamin Dm_0_ antibody and a secondary antibody coupled to a docking strand oligonucleotide. This revealed that Lamin Dm_0_ forms a meshwork of filaments along the inner surface of the nuclear envelope of both nurse cells and follicle cells (Fig. 7A,B), as reported for other cell types (Aebi et al., 1986; Paddy et al., 1990). The nuclear pores cluster in the gaps in the meshwork of Lamin Dm_0_ filaments in both cell types (Fig. 7A,B), consistent with the idea that the Lamins organize the nuclear pores.

To test whether the organization of the nuclear lamina determines the arrangement of the nuclear pores, we mis-expressed the single *Drosophila* A/C-type Lamin (Lamin C) in the follicle cells (Fig. 7C,D) (Riemer et al., 1995). This caused the nuclear pores to become more dispersed, with all Lamin C-expressing cells having lower mean distances between nuclear pore clusters than wild-type cells with similar nuclear pore densities (Fig. 7B,D). Nearly half of the Lamin C-expressing cells fall within the 95% confidence limits for random nuclear pore distributions (Fig. 7E). Lamin C expression also alters the distribution of Lamin Dm_0_, with the Lamin Dm_0_ fibres appearing shorter, thicker and more evenly distributed over the nuclear surface (Fig. 7C).

**Fig. 8.**
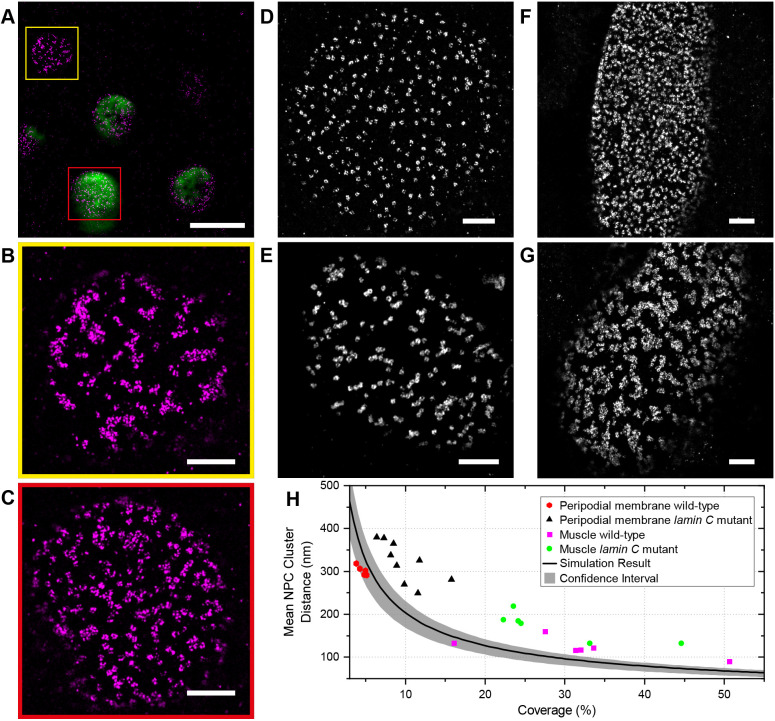
**Lamin C controls NPC positioning.** (A) A DNA-PAINT image of Nup160–Halo (magenta) in a region of the follicular epithelium containing a clone of Lamin C-expressing cells (GFP positive, green) adjacent to wild-type (GFP negative) cells. (B) An enlarged view of the Nup160–Halo distribution in a wild-type nucleus (yellow box in A). The NPCs show normal clustering. (C) Enlarged views of the Nup160–Halo distribution in a nucleus expressing GFP–Lamin C (detail from red box in A). Lamin C overexpression correlates with reduced clustering of the NPCs. (D) A DNA-PAINT image of Nup160–Halo in the nucleus of a wild-type peripodial membrane cell, showing an even distribution of NPC. (E) A DNA-PAINT image of Nup160–Halo in a nucleus of a peripodial membrane from a *lamin C*^EX5^*/Df(2R) trix* mutant larva, showing clustered NPCs*.* (F) A DNA-PAINT image of Nup160–Halo in a wild-type larval muscle nucleus, showing an even distribution of NPCs. (G) A DNA-PAINT image of Nup160–Halo in nucleus from a body wall muscle in a *lamin C*^EX5^*/Df(2R) trix* mutant larva, showing clustered NPCs. (H) A graph showing the mean NPC cluster distance as a function of the proportion of the nuclear envelope covered by NPCs for wild-type and *lamin C* mutant nuclei (D–G). Scale bars: 5 µm (A–C); 1 µm (D–G).

To rule out any background variation in nuclear pore distribution caused by the effects of Lamin C expression on egg chamber development or random differences between individual flies, we induced Lamin C expression in mitotic clones in otherwise wild-type Nup160–Halo egg chambers and compared the arrangement of the nuclear pores in the expressing cells versus adjacent non-expressing cells in the same epithelial layer (Fig. 8A). The nuclear pores are clustered in the wild-type cells, but are more evenly distributed in the Lamin C-expressing cells, confirming that Lamin C acts directly in each nucleus to control nuclear pore positioning (Fig. 8A–C).

The observation that Lamin C expression reduces nuclear pore clustering in cells that do not normally express appreciable levels of the protein suggests that *lamin C* mutants should have the opposite effect on Lamin C-expressing cells. We therefore examined the organization of the nuclear pores in the nuclei of the peripodial membrane of the wing disc in *lamin C*^EX5^/*Df(2R) trix* larvae, which lack all Lamin C (Schulze et al., 2005). Although the nuclear pores are evenly distributed in wild-type peripodial membrane nuclei (Fig. 8D,H), they are organized into clusters or chains in the *lamin C*-null nuclei (Fig. 8E,H)*. lamin C* mutants cause muscle defects in both flies and humans, and we therefore also analysed the nuclear pore distribution in nuclei of the larval body wall muscles (Dialynas et al., 2010; Maggi et al., 2016; Schulze et al., 2009). The nuclear pores are evenly distributed in the nuclear membrane of wild-type muscle nuclei but are highly clustered in the mutant nuclei (Fig. 8F–H). Thus, Lamin C is required for the even spacing of the nuclear pores, demonstrating that the composition and organization of the nuclear lamina controls nuclear pore distribution.

## DISCUSSION

Almost all SMLM imaging to date has been performed on cultured cells that only extend a few micrometres above the coverslip, where one can collect the maximum number of photons with little background signal from out-of-focus light. Here, we describe a simple and cost-effective line scanning microscope system that provides optical sectioning to significantly reduce out-of-focus background in thicker samples, allowing super-resolution SMLM imaging of tissues. Using this system, we routinely obtained images with a resolution of 30 nm or better at depths of more than 20 µm. This extends the superior resolution of SMLM imaging to a much wider range of specimen and cell types.

Our microscope works well for various SMLM techniques, including STORM, but we have focused on developing protocols for performing PAINT imaging, because it is resistant to photobleaching, allows the use of any fluorophore and is very versatile (Jungmann et al., 2014). For example, we have used peptide-PAINT to obtain high-resolution images of the actin cytoskeleton, indirect antibody-mediated DNA-PAINT to visualize the nuclear lamina and direct DNA-PAINT to image endogenous nucleoporins, and we demonstrate that these techniques can be combined for simultaneous two-colour super-resolution imaging. As we generated flies that are homozygous for the tagged proteins, every copy of the protein in the cell carries the tag. This opens up the possibility of extending this approach to count the absolute number of molecules of the protein in any region or structure of interest using qPAINT (Jungmann et al., 2016).

One test of a new imaging approach is whether it allows one to detect features that had not previously been visible. Our SMLM system for imaging tissues has revealed that nuclear pores are not evenly distributed in the nuclear envelopes of most *Drosophila* tissues, as they are in tissue culture cells. Uneven distributions of nuclear pores have been previously observed in several contexts. Mutants in some nucleoporins cause clustering of the nuclear pores on one side of the nucleus, and a similar phenotype is observed in the absence of lamin B and in Hutchinson–Gifford progeria syndrome, which is caused by a truncated form of lamin A/C called progerin (Belgareh and Doye, 1997; Heath et al., 1995; Lenz-Böhme et al., 1997; Liu et al., 2000). Secondly, nuclear pore-free regions are observed in interphase cells shortly after mitosis and might reflect regions of delayed nuclear envelope reassembly (Maeshima et al., 2006). Finally, there is a specific pathway for inserting new nuclear pores as the nuclear envelope grows during interphase and mutants that disrupt this process lead to zones that are free of nuclear pores as the cell cycle progresses (de Magistris and Antonin, 2018; Doucet et al., 2010; Talamas and Hetzer, 2011; Vollmer et al., 2012). Nuclear pore clustering has previously been observed at the interface between the male and female pronuclei of bovine and human eggs immediately after fertilization, where it plays an important role in recruiting the maternal and paternal chromosomes for genome unification (Cavazza et al., 2021). These effects are only observed in specific mutants or cell cycle stages, however, whereas the clustering of nuclear pores in *Drosophila* occurs in wild-type cells, regardless of whether they are still dividing or are postmitotic. More importantly, the non-random distribution of nuclear pores in *Drosophila* occurs on a finer scale than these other phenomena and cannot be detected by confocal microscopy. Thus, the widespread clustering of nuclear pores in somatic cells at all stages of development that we have observed represents a novel organization of the nuclear envelope. Given that some core nucleoporins have been found to associate with specific chromatin domains, NPC clustering could contribute to the internal organization of the nucleus, for example, by recruiting specific chromatin regions to the vicinity of the NPC clusters, thereby facilitating the nuclear export of RNAs transcribed from these regions (Gozalo et al., 2020; Iglesias et al., 2020).

The localization of the nuclear pores negatively correlates with the distribution of the Lamin Dm_0_ and is altered by Lamin C expression or loss, indicating that nuclear pore arrangement is determined by the organization of the nuclear lamina. Biochemical data shows that both B-type and A/C-type Lamins interact with a number of nucleoporins, leading to the suggestion that the lamin meshwork anchors the nuclear pores in place (Hawryluk-Gara et al., 2005; Lussi et al., 2011; Smythe et al., 2000; Walther et al., 2001; Xie et al., 2016). Our results suggest that the B-type Lamin Lamin Dm_0_, does not directly anchor the nuclear pores, but instead corrals them into the spaces between the Lamin filaments. In support of this view, nuclear pores are clustered in lamin B-free regions in mouse adult fibroblasts (MAFs), which only express lamin B (Xie et al., 2016). Furthermore, in both MAFs and *Drosophila* follicle cells, this clustering is lost when A/C-type lamins are expressed. Thus, Lamin C seems to play the critical role in determining an even distribution of nuclear pores, possibly by directly anchoring them to the nuclear lamina.

B-type lamins are constitutively expressed in both *Drosophila* and mammals, whereas A/C-type lamins are expressed in a tissue specific manner (Broers et al., 1997; Riemer et al., 1995). In mammals, the levels of lamin A/C rise with increasing tissue stiffness and these form a meshwork in the nuclear lamina distinct from that formed by lamin B filaments that increases the stiffness of the nuclear envelope, thereby protecting it from mechanical deformation (Lammerding et al., 2006; Swift et al., 2013). It has recently been found that Lamin C expression in *Drosophila* epithelia shows a similar correlation with apical compression (Iyer et al., 2021). Both the peripodial membrane and the folds of the wing disc are under compression and have high Lamin C levels, whereas the wing pouch epithelium, which is not compressed, has low levels. This is mirrored by the distribution of the nuclear pores in each region, with the peripodial membrane and wing folds having an even distribution of pores, in contrast to the wing pouch, which shows clustered nuclear pores. This strengthens the evidence that Lamin C induces an even distribution of nuclear pores and suggests that this is a response to the mechanical state of the tissue. Since the nuclear pores represent gaps in the nuclear envelope, their redistribution may contribute to the effects of Lamin C on nuclear envelope mechanics, for example if dispersing nuclear pore clusters reduces the risk of rupture under stress. Lamin A/C also affects nuclear mechanics by interacting with lamina-associated chromatin domains (LADs), which increases the stiffness of the nuclear envelope and alters gene expression (Harr et al., 2015; Solovei et al., 2013; Stephens et al., 2019; Zheng et al., 2018). These effects could also be mediated in part by the altered distribution of nuclear pores through the association of specific chromatin domains with the NPCs (Gozalo et al., 2020; Iglesias et al., 2020).

Our observations raise the question of whether the clustered organization of nuclear pores that we observe in many *Drosophila* tissues is a more general feature of cells *in vivo*, compared to tissue culture cells. There are very limited data on the distribution of nuclear pores in animal tissues, but it is interesting to note that the nuclear pore arrangement in the follicle cells resembles the ribbons of aligned nuclear pores seen in scanning electron micrographs of Tobacco BY cells (Fiserova et al., 2009). It will therefore be interesting to determine whether this type of nuclear pore organization is common in other species.

## MATERIALS AND METHODS

### Custom-built slit scanning confocal microscope

A shown in Fig. S1, five CW excitation lasers at 405 (Coherent, Obis 405 nm LX), 488 (Coherent, Obis 488 nm LS), 546 (MPB Communications, 2RU-VFL-P-1000-546-B1R), 560 (MPB Communications, 2RU-VFL-P-2000-560-B1R) and 642 nm (MPB Communications, 2RU-VFL-P-2000-642-B1R) are combined with respective dichroic mirrors and pass through an AOTF (AA Opto-Electronics, AOTFnC-400.650-TN) for excitation power control. The diffracted beam (or beams) from the AOTF are coupled into a polarization-maintaining single mode fibre (Thorlabs, PM-S405-XP-Custom). Light exiting the fibre is collimated using a 4× objective lens (Olympus, Plan N 0.10NA; available from Thorlabs as RMS4X) and passes through an adjustable rectangular aperture (Owis, 27.140.0707). The collimated beam passes through a cylindrical lens (Edmund Optics, 68-161). The cylindrical lens focuses the collimated light, along one axis, onto a galvanometer (galvo) mirror (ScanLabs, dynAxis XS 7-1) that is conjugated to the pupil plane of a 100×1.35 NA silicone immersion objective lens (Olympus, UPLSAPO100XS) mounted in an inverted microscope base (Olympus, IX83P2ZF). Excitation light reflecting from the galvo mirror enters the microscope base from the rear port, lower deck, of the microscope base and is reflected into the objective lens with a dichroic mirror (Chroma, 59007bs or ZT405/488/561/647rpc) mounted in the motorized filter turret of the microscope base. The excitation light is focused along one dimension and collimated along the other, when it reaches the objective back pupil plane. The extent (width or diameter) of the light entering the objective back pupil plane was chosen to ensure the pupil is overfilled in the collimated dimension. In this arrangement, the objective lens focuses the excitation light into a line at the sample plane with a diffraction-limited width. The height of the excitation line is set by the rectangular aperture mentioned above. In this way, a line with diffraction-limited width and height of ∼20 µm is created at the sample plane. This line of excitation light is scanned along one dimension, orthogonal to the long axis of the excitation line, by movement of the galvo mirror. Fluorescence emission from the sample is collected by the same objective lens and separated from the laser excitation light with a dichroic mirror mentioned above. After the dichroic mirror, fluorescence emission passes through a multi-channel bandpass filter (Chroma, 59,007 m or ZET405/488/561/647 m) inside the microscope body. Emission light leaves the microscope base via the left side port. An adjustable rectangular aperture (Owis, 27.140.0707) was placed outside the microscope base at the image plane formed by the tube lens. Fluorescence emission was split into two colour channels with a dichroic mirror (Chroma, ZT647rdc). The separated emission light follow identical paths. Each path includes a mirror conjugated to the objective back pupil plane allowing the emission to be slightly offset without losing telecentricity on the detector. The far-red path included a bandpass filter (Chroma, ET705/72 m). The other path included a motorized filter wheel with two filters: one for DAPI/GPF (Chroma, ET525/50 m) the other for Cy3B/ATTO565 (Chroma, ET590/50 m). The separated emission paths were recombined with a dichroic mirror (Chroma, ZT647rdc) and imaged onto an sCMOS camera (Hamamatsu, Orca-Flash 4.0 V3). The effective pixel size on the camera is 98 nm. A slight tilt was added to the respective mirrors in the spectrally separated emission paths such that two images appear next to each other on the camera sensor. The sCMOS camera was operated in light-sheet mode. In this mode, a row of camera pixels is exposed for a predefined exposure time. When that row exposure has finished the next row of pixels is exposed for the same time. This process is repeated such that subsequent rows of pixels are sequentially exposed across a predefined region of interest (ROI). The per row exposure time may be user set and, independently, how quickly new rows are exposed can also be user set. In this way, the number of adjacent rows simultaneously being exposed can be controlled by the user. Limiting the camera exposure to a set number of rows is equivalent to using a mechanical (confocal) slit to limit out-of-focus background from reaching the detector (Mei et al., 2012). As the exposed rows sequentially progress along the camera sensor, the glavo mirror synchronously scanned the excitation line across the sample. Synchronization between the galvo mirror and sCMOS camera was carried out with a data acquisition card (National Instruments, PCIe-6343) and custom software written in the LabView environment (National Instruments, 64 bit, LabView 2016). All aspects of microscope control and data acquisition were carried out using a custom microscopy hardware control program developed in the LabView environment and freely available on GitHub (https://github.com/Gurdon-Super-Res-Lab/Microscope-Control).

### Sample drift correction during acquisition

Unlike cultured cells, *Drosophila* egg chambers do not tightly adhere to a slide or cover glass surface and the desired focal plane is typically many micrometres above the cover glass. These two complications preclude using the two of the most common sample drift correction methods: (1) tracking the axial cover glass position with a reflected far red beam and (2) attaching fiducial markers to the cover slip for lateral drift correction in post processing. Thus, following from the work of McGorty and co-workers (McGorty et al., 2013), we implemented a ‘real-time’ drift correction scheme based on transmitted light images of the sample itself. The microscope was modified (Fig. S1) to include a near-IR light emitting diode (LED) lamp (Thorlabs, M850LP1) for transmitted light imaging of the sample outside the fluorescence detection channel. An additional short-pass dichroic mirror (Chroma, ZT775sp-2p) was placed just below the microscope objective to reflect this light through an emission filter (Thorlabs, FL780-10) and tube lens (Thorlabs, AC254-100-B), and onto an inexpensive sCMOS camera (Edmund Optics, 84-933). In this way, transmitted light images were collect in parallel with fluorescence images. Prior to starting fluorescence image acquisition, a *z*-stack of 11 transmitted light images spaced 100 nm apart in *z*, centred around the initial sample position, were collected using the lamp and camera as described above. The central and two extreme images were marked as reference images and used to calculate a dimensionless parameter *ξ* following from McGorty (McGorty et al., 2013) as *ξ*_*n*_=(*PV*{*C*_+,*n*_}−*PV*{*C*_−,*n*_})/*PV*{*C*_0,*n*_} where *PV*{*C*_+,*n*_} is the peak correlation value between the current image and the upper most reference image, *PV*{*C*_−,*n*_} is the peak correlation value between the current image and the bottom most reference image and *PV*{*C*_0,*n*_} is the peak correlation value between the current image and the central reference image. The stack of reference images was used to generate a z-position vs *ξ* curve, which was fitted with a linear function for calibration. During imaging, a transmitted light image was collected at 1.25 Hz and used to compute *ξ*_*n*_. If the sample *z*-position changed by more than ±20 nm, the sample z-piezo stage was moved to correct the drift. Although this method allows the position of the sample to be tracked in three dimensions (3D), stepper motor driven sample *xy* stages do not allow small enough repeatable steps to make *xy* correction reliable while imaging. Thus, a hybrid approach was employed for lateral drift correction. While imaging, the sample *z*-position was actively tracked and corrected using the piezo sample *z*-stage (Märzhäuser, 00-55-550-0800). SMS (super resolution) camera frames were collected in blocks (cycles) of 1000. After each cycle (1000 frames) the acquisition was paused and if the sample was found to have laterally drifted more than a user pre-set amount, the sample *xy*-stage would be moved to correct the drift and then imaging resumed.

### Optimizing imaging parameters

The excitation laser intensity is an important parameter with a profound effect on the quality of super-resolved images (Diekmann et al., 2020). Within the context of line scanning DNA-PAINT, we optimized excitation intensity to increase the number of photons per localization without negatively impacting the labelling efficiency due to photodamage. Although DNA-PAINT is resistant to photobleaching due to the abundance of free-floating dyes attached to imager strands, docking strands are not exchangeable and are susceptible to photodamage (Blumhardt et al., 2018). To avoid depleting docking strands from photodamage we monitored the number of localizations per frame. For ATTO 643 and Cy3B, we empirically found that a diffraction limited excitation line with less than 2 mW average power maintained a reasonably constant number of localizations throughout the course of imaging as shown in Fig. S7A,B. Using this excitation level, the electronic slit width was varied. A slit width of 16 lines (8 lines=1 airy unit) was found to produce the best localization precision (number of photons versus background rejection; Fig. S7C).

### SMLM image reconstruction

Single-molecule events on unprocessed camera frames were fit with a symmetric Gaussian model PSF as described previously and available in a single-molecule fitting package from Li et al. (2018). Fit data was filtered based on localization precision (0.5–30 nm), PSF width (50–150 nm), and log-likelihood. The filtered fit data was drift corrected using the algorithm from Wang and co-workers (Wang et al., 2014). The MatLab drift correction function available on the author's GitHub page (https://github.com/yinawang28/RCC) was used with slight modifications to work with the fit data from the analysis pipeline used herein.

### Two-colour image registration

Two-colour images were registered using the method previously described by Huang and co-workers (Huang et al., 2016). Tetraspeck beads immobilized on glass and mounted in index-matched medium were raster scanned across the microscope field of view. At each step in the scan, an image was recorded in both colour channels. Beads positions in each recorded frame were localized and the (*x*,*y*) positions saved. Corresponding bead positions in each colour channel were used to generate a transformation matrix that was used to register our two colour images. Typical registration error between bead images was less than 5 nm. Image registration calibration was performed daily.

### Reagents

DNA oligonucleotides (conjugated to fluorophores) were purchased from Integrated DNA Technologies or AtdBio. DNA oligonucleotides conjugated to SNAP or Halo tag ligands were purchased from AtdBio or Biomers.net. A non-commercial modified version of Halo ligand (PBI 300-43) was a gift from Mark McDougall (Custom assay services, Promega corporation, USA).

### Antibodies

Antibodies used were: mouse monoclonal anti-Lamin Dm0 (DHSB, ADL84.12, used at 1:200), mouse monoclonal anti-α-Tubulin−FITC (Merck, F2168, used at 1:500), Massive SDAB 1-Plex (Massive Photonics, used at 1:200); and the Massive Tag-q Anti-GFP Kit (Massive Photonics, used at 1:200).

The results of the commercial antibodies below are consistent with the information shown on respective manufacturers’ websites, where references are listed.

### *Drosophila melanogaster* lines

Single-channel actin images were taken using *w*^1118^ flies. For imaging of nucleopore proteins, several endogenously-tagged lines were created. Nup188, Nup160 and Gle1 were tagged at the C-terminus with SNAP and Halo tags using CRISPR/Cas9 mediated homologous recombination. Guide RNA sequences were identified using http://tools.flycrispr.molbio.wisc.edu/targetFinder/, and cloned into the pCFD3 plasmid for guide RNA expression (Port et al., 2014). Table S2 lists the guide sites used. Around 1 kb of sequence upstream and downstream of the gRNA sites were cloned into the EcoRI and NotI sites of pBluescript SK+ (Stratagene) along with either the SNAP- or Halo-coding sequence, and a short linker sequence, using NEB Hi-Fi assembly mix (New England Biolabs). A mixture of the two guide plasmids (each at 100 ng/µl) and homologous repair plasmid (150 ng/µl) was injected into CFD2 embryos (Port et al., 2014), which express Cas9 under the *nanos* promoter. Resulting F0 adults were screened by PCR to identify potential knock-ins, positives were balanced and stocks created.

UAS-GFP-*lamin C* was a gift from Prof. Kazuhiro Furukawa, Niigata University, Japan (Uchino et al., 2013). UAS-GFP-*lamin C* was expressed under the control of Gr1-Gal4. Clones of GFP-*lamin C*-expressing follicle cells were generated by crossing *y w*; *hs-FLP*; Act5C>CD2>Gal4, UAS:mRFPnls (Bloomington *Drosophila* Stock Center, 30558) to UAS-GFP- *lamin C. lamin C* ^EX5^ (a gift from Prof. Lori Wallrath, University of Iowa, USA) and *Df(2R) trix*/*CyO* (BDSC*,*1896) were both recombined with Nup160–Halo and used to generate *lamin C* mutant third-instar larvae.

### *Drosophila* genetics and dissection of tissues

Standard procedures were used for *Drosophila* maintenance and experiments. Follicle cell clones of Lamin C were induced by incubating hs-FLP; FRT mutant marker adults at 37°C for 2 h, which were then dissected after two days. All flies were fattened on yeast at 25°C for 1 to 2 days before dissection. Ovaries were dissected in Schneider's medium at room temperature and the muscle sheath surrounding the egg chambers was removed. Testis accessory glands and ejaculatory ducts were dissected from adult males, wing discs and body wall muscles were dissected from wandering third instar larvae.

Embryos from 1.5–2 h collections on apple juice plates were dechorionated with 50% bleach for 3–5 min, and washed three times with water. Embryos were fixed for 20 min in 3% formaldehyde in 0.5× PBS overlaid with heptane (1:1 fixative/heptane). Embryos were transferred to double-sided tape stuck to the base of a 100 mm petri dish, containing 0.2% Tween 20 in PBS. The vitelline membrane was removed using a syringe needle.

### Peptide-PAINT fixation and staining

Ovarioles were dissected out of the muscle sheath, and warm (38°C) fixation buffer was added (4% methanol-free formaldehyde, 2% Tween 20 in 0.5× PBS). Samples were fixed for 20 min at room temperature with rotation. Samples were washed 3×10 min with 0.2% Tween 20 in PBS.

Egg chambers were attached to a coverslip (high precision) using Cell-Tak (Corning, 354240) and then mounted on concavity slides in ∼150 μl of labelling solution {0.5–5 nM Cy3B Lifeact in PBS with either 20 mM sodium sulphite or PCA (3,4 dihydroxybenzoicmacid), PCD (protocatechuate 3,4-dioxygenase pseudomonas), Trolox [(+−)-6-hydroxy-2,5,7,8-tetra methylchromane-2-carboxylic acid]; Table S2}. Slides were sealed with two-compound silicone glue and kept in the dark for 5–10 min until the glue solidified. Alternatively, egg chambers were attached to the coverslip base of an 8-well chambered cover glass with Cell-Tak, and ∼200–300 µl labelling solution was added. N-terminally labelled Cy3B Lifeact was synthesized by Peptide Protein Research Ltd. Mounting methods, imaging solutions and imaging conditions are indicated in Table S3.

### DNA-PAINT fixation and staining

For egg chamber samples with endogenously Halo- or SNAP- tagged proteins, ovarioles were fixed in 3% formaldehyde in 0.5× PBS for 15 min at room temperature. Samples were washed 3×5 min in PBS, then permeabilized in 0.5% Triton X-100 for 5 min. They were then quenched with 50 mM ammonium chloride in PBS for 5 min, washed for 5 min in PBS and incubated in Image-iT FX signal enhancer (Thermo Fisher Scientific, I36933) for 30 min. The labelling reaction was performed for 1 h at 37°C, with shaking, in 0.5% bovine serum albumin (BSA), 1 µM DTT in PBS with 1 µM of docking oligonucleotide. Samples were washed 3×5 min in PBS followed by 0.1% Triton X-100 in PBS overnight. The following day samples were washed 6×10 min in PBS before being mounted for imaging. Egg chambers were attached to an 8-well chambered cover glass with Cell-Tak, and ∼200–300 µl imaging solution containing fluorescently labelled imager strand, and oxygen scavengers. Mounting methods, imaging solutions and imaging conditions are indicated in Table S3.

To combine DNA-PAINT with Lifeact imaging, the protocol was followed as above, but 0.5–2 nM Cy3B Lifeact peptide was added to the imaging solution.

To perform two-colour DNA-PAINT imaging of a SNAP or Halo-tagged protein and a GFP-tagged protein, the latter was labelled with a GFP-nanobody coupled to a docking strand oligonucleotide (Massive Photonics, Massive Tag-q Anti-GFP Kit). The same protocol was followed as above, with the GFP-nanobody incubated with the sample along with the SNAP/Halo docking strand.

To perform DNA-PAINT with a primary antibody followed by a secondary antibody conjugated to the docking strand, the egg chambers were blocked with 10% BSA (in PBS with 0.2% Tween 20) for 1 h at room temperature after fixation and permeabilization. The samples were then incubated with primary antibodies against Lamin Dm_0_ (1:200) in PBS with 0.5% BSA at 4°C overnight. After 6×10 min washes in PBS with 0.1% Tween 20, egg chambers were quenched with 50 mM ammonium chloride for 5 min. The following steps were the same as the method of single DNA-PAINT described above, except that the secondary antibody conjugated to the docking strand (Massive SDAB 1-Plex, Massive Photonics) was incubated along with the SNAP/Halo docking strand.

For the testis accessory gland, wing discs and body wall muscles, the tissues were fixed with 4% formaldehyde, 2% Tween in 0.5× PBS for 20 min at room temperature. Samples were then washed 3×5 min in PBS and then treated with 50 mM ammonium chloride in PBS for 5 min. All subsequent steps were the same as for egg chambers, except that the labelling reaction was performed for 2 h at 37°C, and all steps were undertaken in glass dishes, rather than 1.5 ml tubes.

### DNA-PAINT on Nup96–Halo U2OS cells

Cells were seeded in a 35 mm imaging dish with a glass bottom 24 h before the labelling, so that they reached 50% confluency the next day. Cells were prefixed for 30 s in 2.4% (w/v) PFA in PBS and then permeabilized for 3 min in 0.4% (v/v) Triton X-100 in PBS. Cells were then fixed for 30 min in 2.4% (w/v) PFA in PBS and incubated for 5 min in 100 mM NH_4_Cl in PBS. Cells were then washed 2×5 min in PBS and incubated for 30 min in Image-iT FX signal enhancer. The labelling reaction was performed for 2 h at room temperature 1 µM docking strand with 1 µM of DTT in 0.5% BSA in PBS. After the labelling, the cells were washed for 3×5 min in PBS and imaged in imaging solution. U2OS cell lines expressing Nup96–Halo were a kind gift from the Ries laboratory (EMBL, Heidelberg, Germany; Thevathasan et al., 2019). The Nup96–Halo labelling was authenticated, but no further authentication was performed. Cell lines were not tested for mycoplasma contamination.

### Buffers

100× PCD and 40× PCA were made according to Schnitzbauer and co-workers (Schnitzbauer et al., 2017).

100× Trolox was prepared either according to Schnitzbauer and co-workers (Schnitzbauer et al., 2017), or dissolved directly in methanol to give a 100 mM solution, as per Cordes and co-workers (Cordes et al., 2009), which was diluted to 1 mM in imaging solution and aged using UV exposure to give ∼125 µM Trolox-quinone and ∼875 µM Trolox before use. This procedure ensured a consistent balance between the oxidizing and reducing capacity of the Trolox. In our hands, 2 min of exposure to UV on a gel-doc transilluminator produced the desired ratio.

The basic imaging solution for DNA-PAINT was 1× PBS pH 7.2, 500 mM NaCl, and that for Peptide**-**PAINT was 1× PBS pH 7.2 500 mM NaCl, or 1× PBS pH 7.2.

### Drug treatments and immunofluorescence staining

To depolymerize actin and tubulin, egg chambers were treated with 100 μM Latrunculin A (Abcam) and 50 mg/ml colcemid (Santa Cruz Biotechnology), respectively, in Schneider's medium for 1.5 h at 25°C. Control egg chambers were incubated in Schneider's medium for 1.5 h at 25°C. Egg chambers were fixed in 4% formaldehyde, 2% Tween 20 in PBS for 20 min and then washed 3×5 min in 0.1% Tween in PBS. Egg chambers were then incubated with a 1:500 dilution of Phalloidin-iFluor 405 (Abcam) and anti-α-Tubulin−FITC antibody (Merck) in PBS, 0.5% BSA at 37°C for 1 h with shaking. Samples were washed for 6×10 min in 0.2% Tween in PBS buffer, mounted in Vectashield with DAPI (Vector Laboratories) and examined via confocal microscopy (Leica SP8).

### Live imaging of Nup160–Halo

Egg chambers were dissected out of the muscle sheath and incubated in Schneider's medium containing 0.5 µM JF646–Halo for 20 min. The egg chambers were then removed to Schneider's medium and imaged in an 8-well µ-slide with poly-lysine coating (Ibidi) using a Zeiss Airyscan microscope.

### Morphological image analysis

#### Image binarization

Circular regions of interests from greyscale super-resolved images were binarized using an adaptive threshold based on the gaussian weighted mean intensity of an 11-pixel neighbourhood around each pixel. For Nup160 images, isolated and spur pixels were removed from the resulting binary image along with foreground areas with sizes smaller than half the expected area of an NPC. Next, the binary image was morphologically closed with a 2-pixel square structuring element, followed by an opening operation with the same structuring element. Finally, areas of the binary image where the mean intensity in the corresponding grayscale image was less than 10% of the maximum grayscale value were considered nonspecific signal and removed. Results for a variety of coverage scenarios ranging from low to high coverage are presented in Fig. S8A–A‴.

For Gle1 images (Fig. S8B,B′), isolated and spur pixels were removed after binarization, along with areas of the binary image where the mean intensity in the corresponding grayscale image was less than 10% of the maximum grayscale value. Finally, the binary image was morphologically closed with a 5-pixel radius disc structuring element, followed by a dilation with a 4-pixel radius disc structuring element.

#### Estimating mean cluster distance

Binary images were Euclidean distance transformed (Fig. S8C–C′). To select only the pixel values along the ridges, whose values correspond to the half-distance between clusters, a morphological thinning operation was performed to produce a skeleton of the binary image (Fig. S8C″). A round mask was also applied to exclude edge and peripheral pixels (Fig. S8C″). The mean of the grayscale pixel values in the distance transformed image traced by the skeleton was multiplied by 2 to obtain an estimate on the mean cluster distance in pixels (1 pixel corresponds to 10 nm).

#### Estimating coverage

For the purpose of this work, a binary image of a single NPC should appear as a disc with a radius of 5 pixels (50 nm). However, image binarization tends to overestimate the size of large NPC clusters (Fig. S8D,E). To correct this, we analysed single NPCs and estimated the effective radius assigned by the binarization process. For data collected in the line scanning setup, a circular disc with an effective radius of 7.8 pixels was found to represent the area assigned to a single NPC indicating that the error introduced in the coverage by the binarization process is that of a circular ring with an inner ring radius 2.8 pixel smaller than its outer radius. The correction on the effective radius for the data collected in the TIRF system was 3.6 pixels.

For more complicated geometries associated with clusters of several NPCs and non-circular shapes, we calculated the radius that would correspond to a disc with the same area as the cluster under consideration. We applied the appropriate correction on this radius. The area calculated using the corrected radius was used for further data analysis.

#### Simulation of NPC super-resolved images

To analyse the spatial distribution of NPCs, we began with a test of the complete spatial randomness (CSR) hypothesis using the Clark–Evans method (Clark and Evans, 1954) on simulated images. We found that this method is more sensitive to the local characteristics of the NPC distribution, which, in this case, is dominated by the condition that the NPCs cannot overlap, and its ability to detect more global characteristics of the NPC distribution is compromised. This behaviour is not surprising given that the Clark–Evans test relies on nearest neighbour distances. Alternative approaches to test the CSR hypothesis at longer distances, such as the Ripley's K function method (Ripley, 1977), require the location of every NPC on the nuclear surface which, under our experimental conditions, cannot be determined accurately. However, we can determine with higher accuracy the regions of the nuclear membrane that are covered with NPCs and we developed a method to obtain a baseline for comparing the NPC cluster distribution between different tissues. We generated simulated NPC images by randomly distributing NPCs, with the condition that NPCs cannot overlap, and varied the number of NPCs to achieve coverages from 3–60%. For each coverage condition, datasets with a labelling efficiency ranging from 20–100% were simulated (Fig. S4). To factor in variability associated with the random distribution of NPCs, 10 repeats for each coverage and labelling condition were performed. The simulated images were morphologically analysed in an identical way to the super-resolved images obtained from the different tissues.

The mean NPC–cluster distance versus coverage for the different labelling conditions and corresponding repeats is shown in Fig. S4D. Based on these data an expected trend is drawn by fitting the 50% label efficiency data with a power-law (a*x^*b*^). Upper confidence intervals are estimated by selecting only the highest NPC–cluster distance value at each coverage value and fitting those values with the same power-law. Lower confidence intervals are calculated in a similar way, but by selecting the corresponding lowest NPC–cluster distance at each coverage value.

### SMLM image feature fitting

In all instances, rather than using a line profile with pixel intensities from the reconstructed image we have used the underlying (*x*,*y*) localization data. Specifically, reconstructed images were annotated with line ROIs (user drawn line segments). The corresponding (*x*,*y*) localizations within a fixed distance (±300 nm) from a line ROI were selected and the (*x*,*y*) midpoint of the line was subtracted from the selected points such that they were centred at the origin. The shifted (*x*,*y*) localizations were transformed to polar (*r*, θ) coordinates and the line ROI's angle was added to the θ component. The localization coordinates were transformed back into cartesian (*x*,*y*) coordinates. The shifted and rotated (*x*,*y*) coordinates were binned along the horizontal axis to produce a profile that is equivalent of summing localization along the original line ROI. The binned localizations were fitted with a single (double) Gaussian to determine the width (distance between) the feature(s).

## Supplementary Material

Supplementary information
