# Supplementary material for: A single-molecule localization microscopy method for tissues reveals nonrandom nuclear pore distribution in Drosophila
Source: J Cell Sci. 2021 Dec 16;134(24):jcs259570. doi: 10.1242/jcs.259570 (PMC8729783; doi:10.1242/jcs.259570)
Supplement: Supplementary information [file joces-134-259570-s1.pdf]

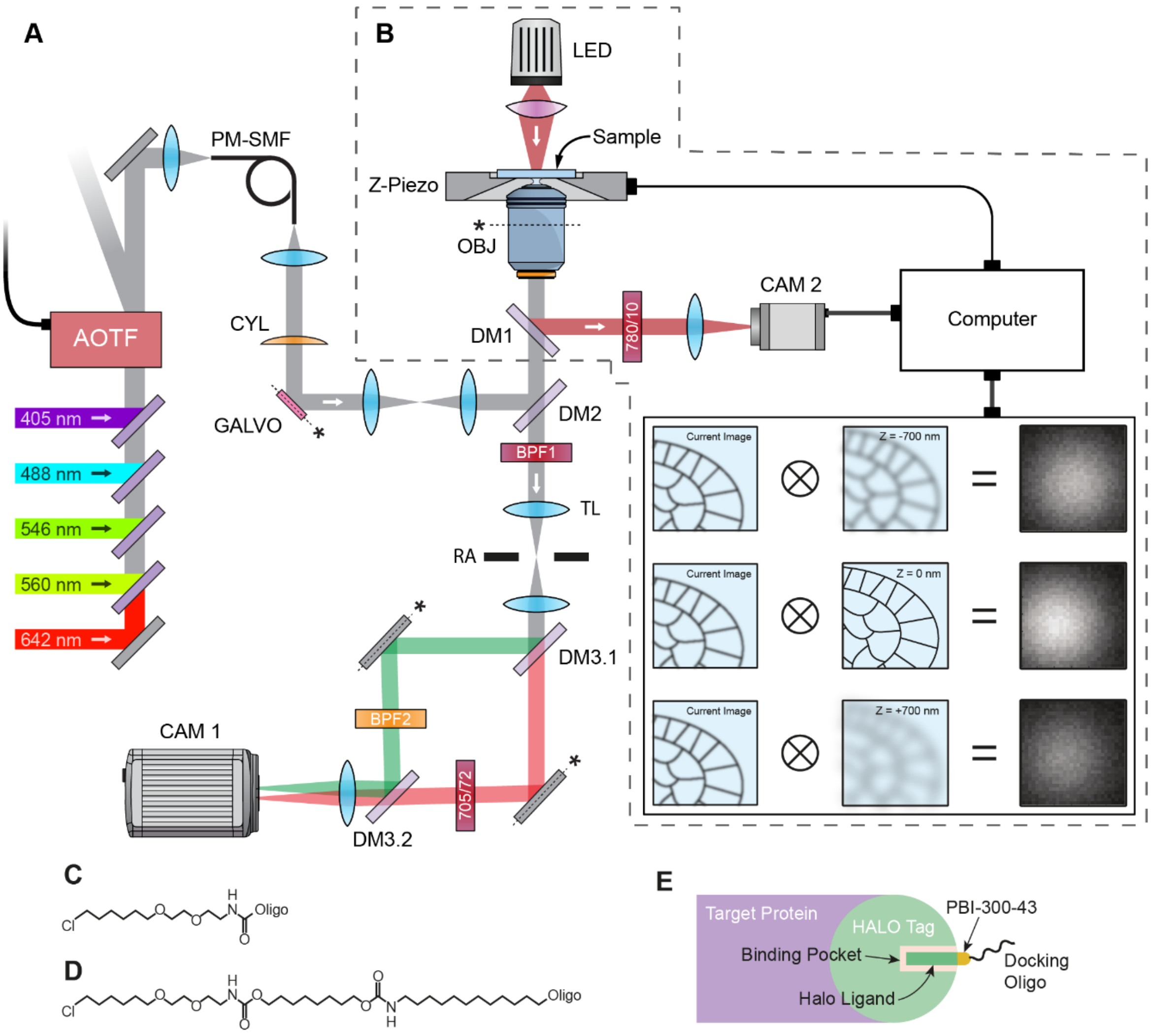

Fig. S1. Line Scanning Confocal Microscope Setup. (A) Five excitation lasers are coupled into a fibre (PM-SMF), collimated at the fibre exit, and pass through a cylindrical lens (CYL) that focuses the beam in one dimension onto a galvanometer mirror (GALVO). The excitation laser light is reflected by a dichroic mirror (DM2) into the objective lens (OBJ) where it forms a line of excitation light at the sample plane. Fluorescence is collected by the same objective lens, separated from the laser excitation light by DM2, passes through a bandpass filter (BPF1) and is imaged by the microscope base's tube lens (TL). A rectangular aperture (RA) is placed at the microscope image plane. The fluorescence image is relayed by a telescope onto the primary imaging camera (CAM1). Fluorescence is split into two colour channels with a dichromic mirror (DM3.1), slightly tilted using the subsequent fold mirrors and recombined using a second dichromic mirror (DM3.2). The far-red beam path includes a fixed bandpass filter (705/72) while the alternate beam path includes a motorized filter wheel for switching between GFP and Cy3B (BPF2). **(B)** Far-red 850 nm light is emitted from an LED lamp (LED) mounted above the sample. The LED lamp light is collected by the objective lens and separated from the fluorescence beam path with a dichroic mirror (DM1). Light reflected from DM1 passes through a bandpass filter (780/10) and is focused by a 100 mm focal length lens onto a USB camera (CAM2). The real-time, or current image, collected by CAM2 is cross correlated with previously collected reference images at, above and below the focal plane to track the (x, y, z) sample position. The computer uses the cross-correlation information to correct for any sample Z drift by adjusting the position of the sample pizeo z-stage (Z-Piezo) during image acquisition. **(C)** Schematic of a typical Halo ligand. **(D)** Halo ligand modified with PBI-300-43. **(E)** Geometry of Halo tag and oligo docking strand used for DNA-PAINT.

Journal of Cell Science • Supplementary information

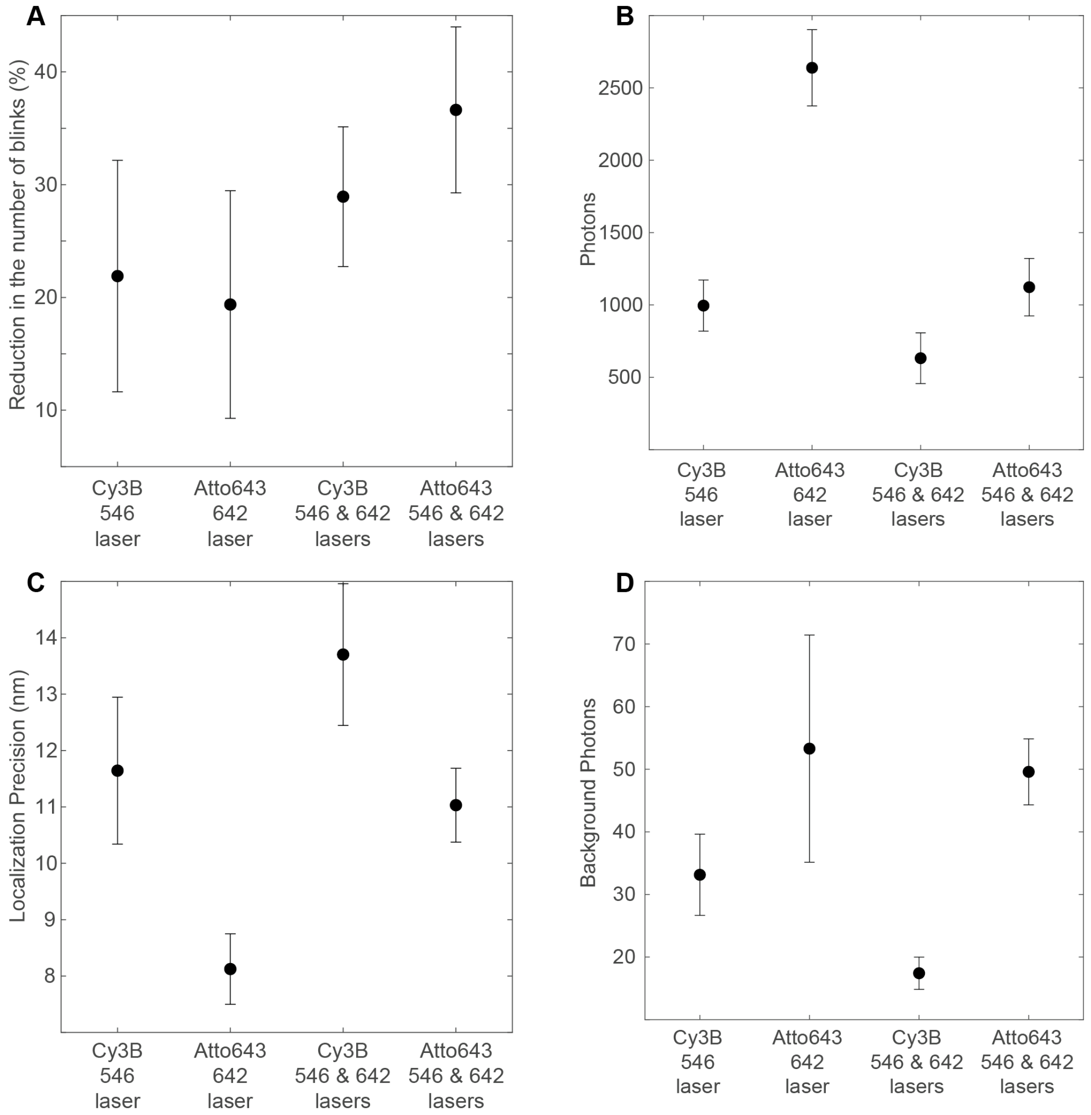

Fig. S2. Representative experimental localization imaging results. (A) Reduction in the number of blinks detected between the first 1000 frames and the last 1000 frames in an acquisition. **(B)** Mean number of photons. **(C)** Mean localization precision. **(D)** Mean number of background photons. x-axis values indicate the fluorophore and excitation lasers used during imaging. Error bars indicate the mean ± the standard deviation (n = 6 for each laser and probe combination).

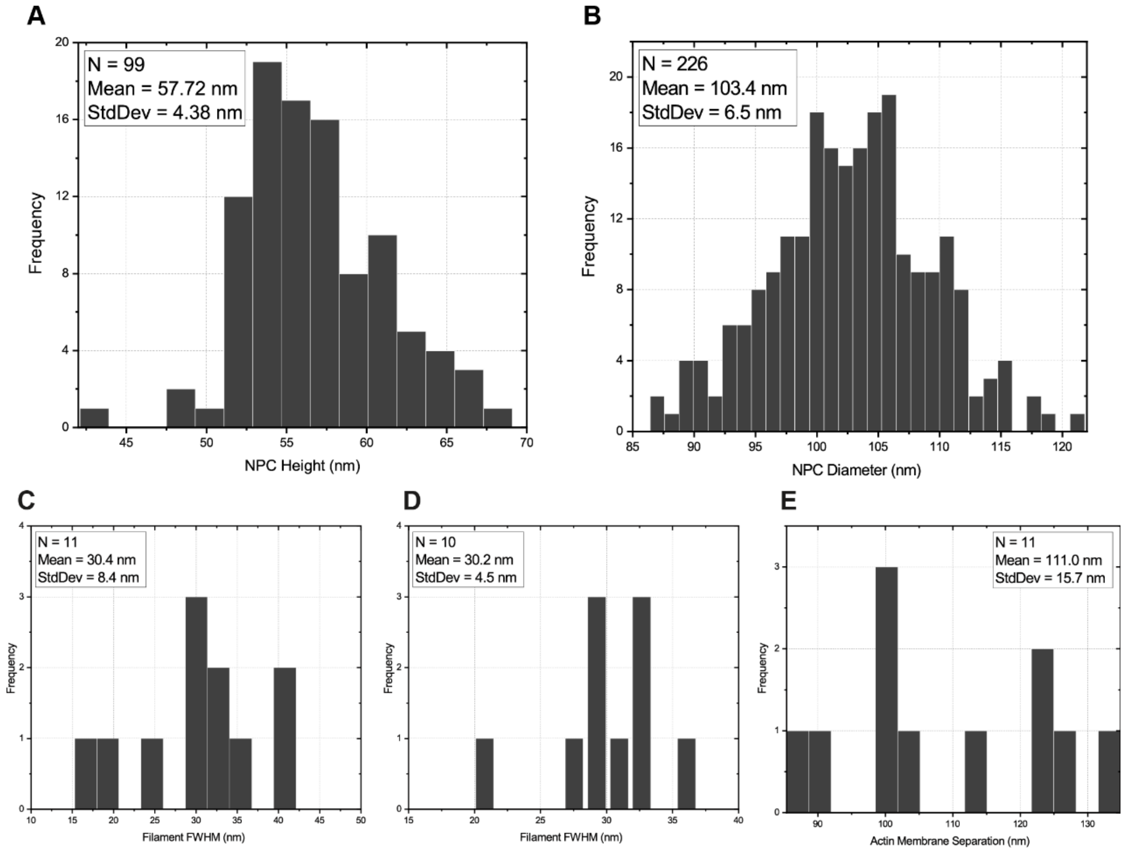

Fig. S3. The distributions of NPC and actin filament widths. (A) 99 NPCs cross-section ROIs were selected from 21 separate Nup160 images. For each ROI, the (x, y) localizations were rotated such that the long dimension of the NPC rings was parallel with the horizontal axis, and summed along the horizontal axis to generate a histogram with position on the x-axis and number of localizations on the y-axis. These data were then fitted with two Gaussians and the distance between the fitted peak positions was determined. **(B)** 226 ROIs containing a single, spatially separated, NPC were hand selected from 27 separate Nup160 images. The (x, y) localizations in each ROI were filtered to remove any (x, y) point that was more than σ/8 away from its nearest neighbour. The remaining (x, y) points were fit with a circle to determine the NPC diameter. **(C)** 11 basal actin filaments were selected, binned along their long axis, and fit. The mean fitted FWHM was 30.4 ± 8.4 nm (mean ± s.d.) **(D)** 10 apical actin filaments were selected, binned along their long axis, and fit. The mean fitted FWHM was 30.2 ± 4.5 nm (mean ± s.d.) **(E)** 11 locations showing actin cortices in adjacent cells were selected across 7 images. For each location, a line was drawn between the parallel membranes and localizations around the marked lines were segregated, binned, and fit with a double Gaussian function to determine the separation between the two membranes as 111.0 ± 15.7 nm (mean ± s.d.).

Journal of Cell Science • Supplementary information

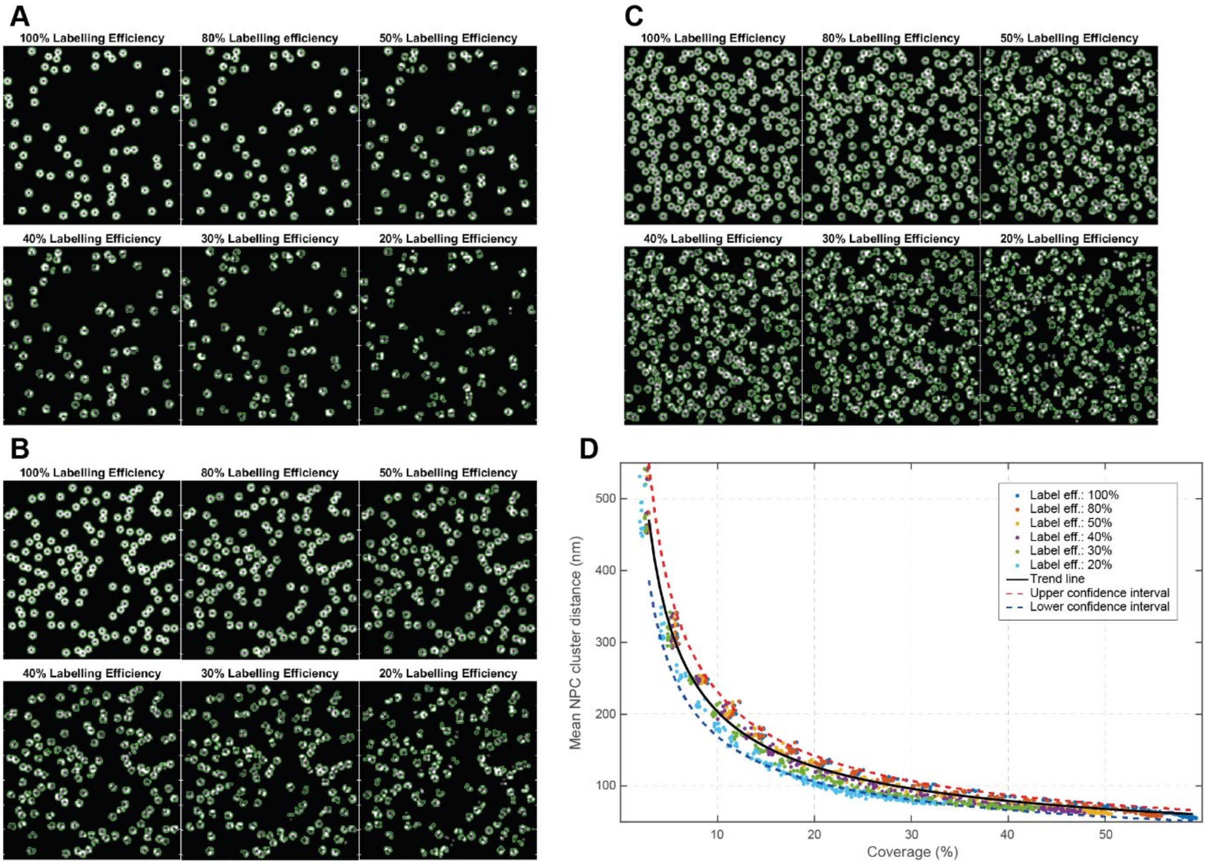

Fig. S4. NPC simulation and analysis results (A) Overview of simulated NPC datasets with 6% coverage and different levels of labelling efficiency. **(B)** Overview of simulated NPC datasets with 12% coverage and different levels of labelling efficiency. **(C)** Overview of simulated NPC datasets with 24% coverage and different levels of labelling efficiency. **(D)** Mean NPC-cluster distance vs Coverage.

Journal of Cell Science • Supplementary information

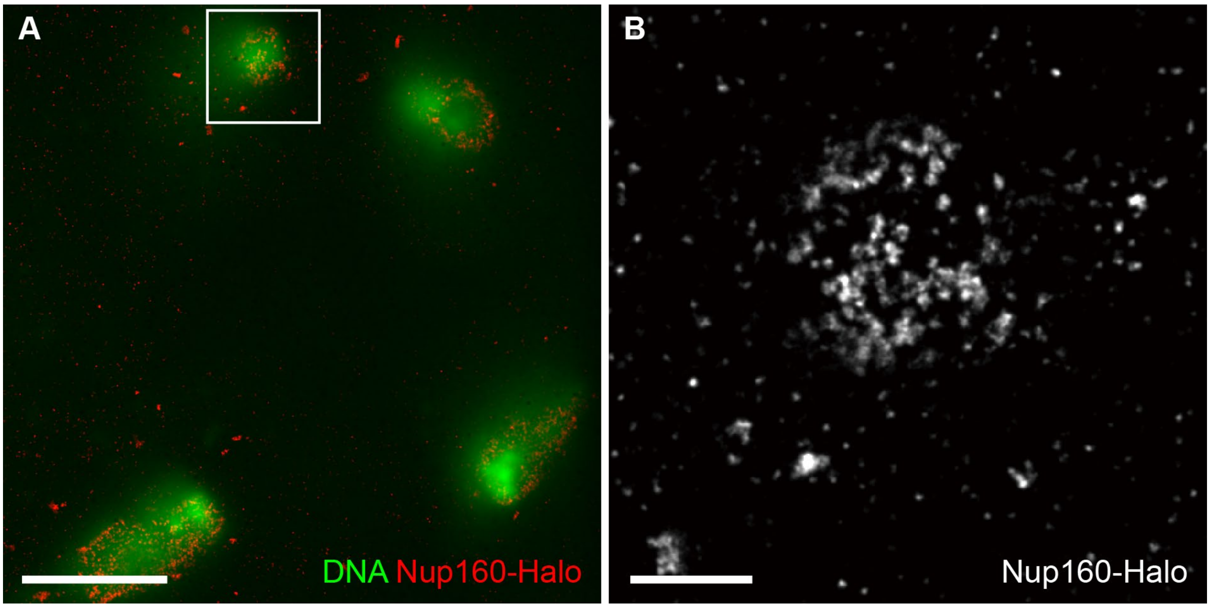

Fig. S5. NPC clustering is present in syncytial blastoderm nuclei immediately after mitosis. (A) A combined confocal and DNA-PAINT image of DNA (DAPI; green) and Nup160-Halo (red) in a nucleus that has recently divided in a syncytial blastoderm embryo, showing clustered NPCs. Scale bar, 5 µm. **(B)** A magnified view of the boxed region in **(A)** showing NPCs labelled with Nup160-Halo. Scale bar, 1 µm.

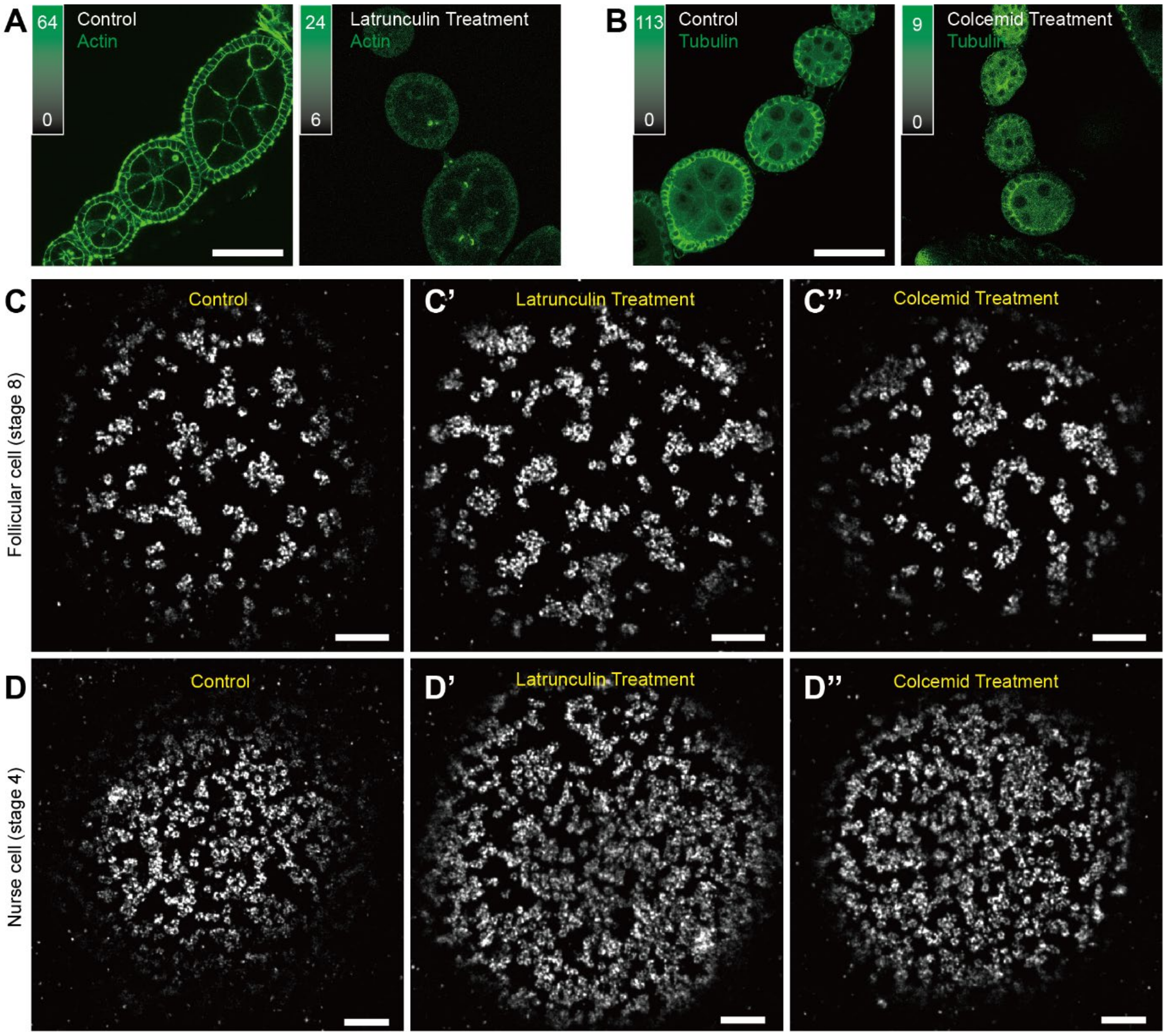

Fig. S6. NPC clustering is unaffected by actin or microtubule disruption. Actin and microtubules were disrupted by treatment with Latrunculin **(A)** and Colcemid **(B)**, respectively, as shown when imaged with confocal microscopy. In stage 8 *Drosophila* egg chamber, The NPCs **(C)** in stage 8 follicle cells remain clustered when treated with Latrunculin **(C')** and Colcemid **(C'')** and imaged using DNA-PAINT. Similarly, the NPCs **(D)** remain clustered in stage 4 nurse cells when treated with Latrunculin **(D')** and Colcemid **(D'')**. Thus, NPC clustering is not dependent on actin or microtubules. Scale bars are 5 µm for panels **(A)** and **(B)**, and 1 µm for panels **(C)** and **(D)**.

Journal of Cell Science • Supplementary information

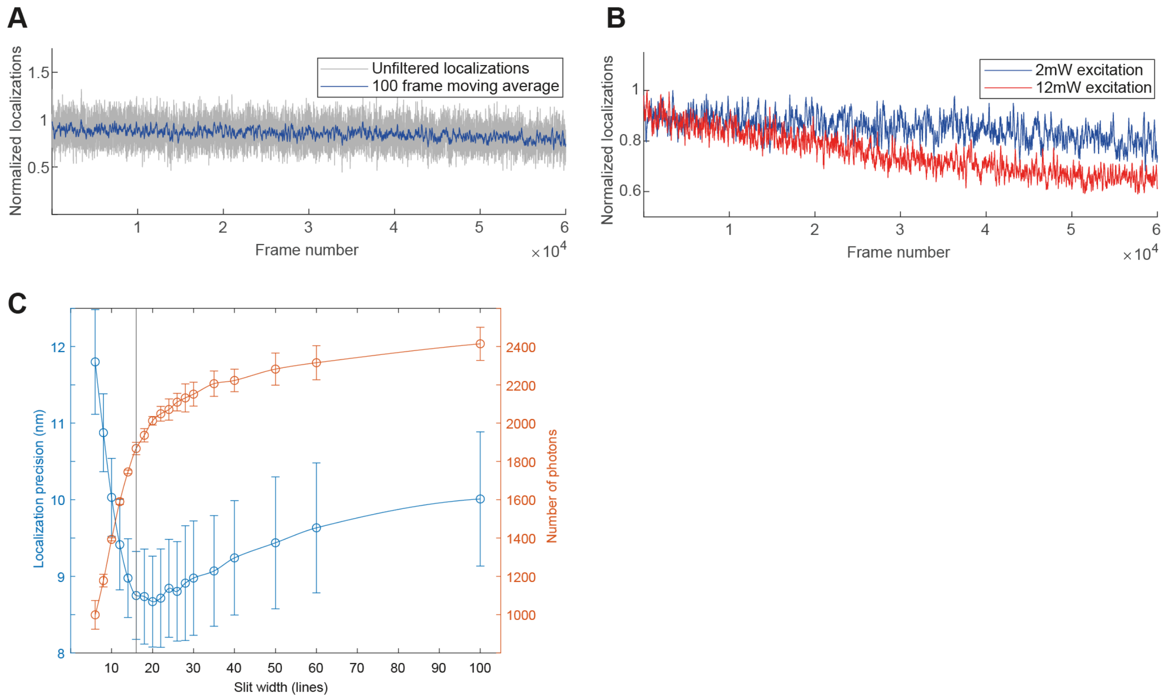

Fig. S7. Optimization of imaging parameters: (A) Number of localizations as a function of frame number. Gray trace indicates the raw number of localizations and the blue trace the moving average over 100 frames under 2mW excitation and 4Hz framerate. **(B)** 100-frame moving average of localizations as a function of frame number under 2mW excitation (blue trace) and 12mW excitation (red trace) recorded at 4Hz framerate. **(C)** Mean localization precision (blue trace) and mean number of photons (red trace) as a function of slit width of 500 frames under 20% excitation recorder at 4Hz frame rate in the presence of 1nM Atto643 imager strand solution. Error bars indicate mean ± s.d. for 3 independent experiments.

Journal of Cell Science • Supplementary information

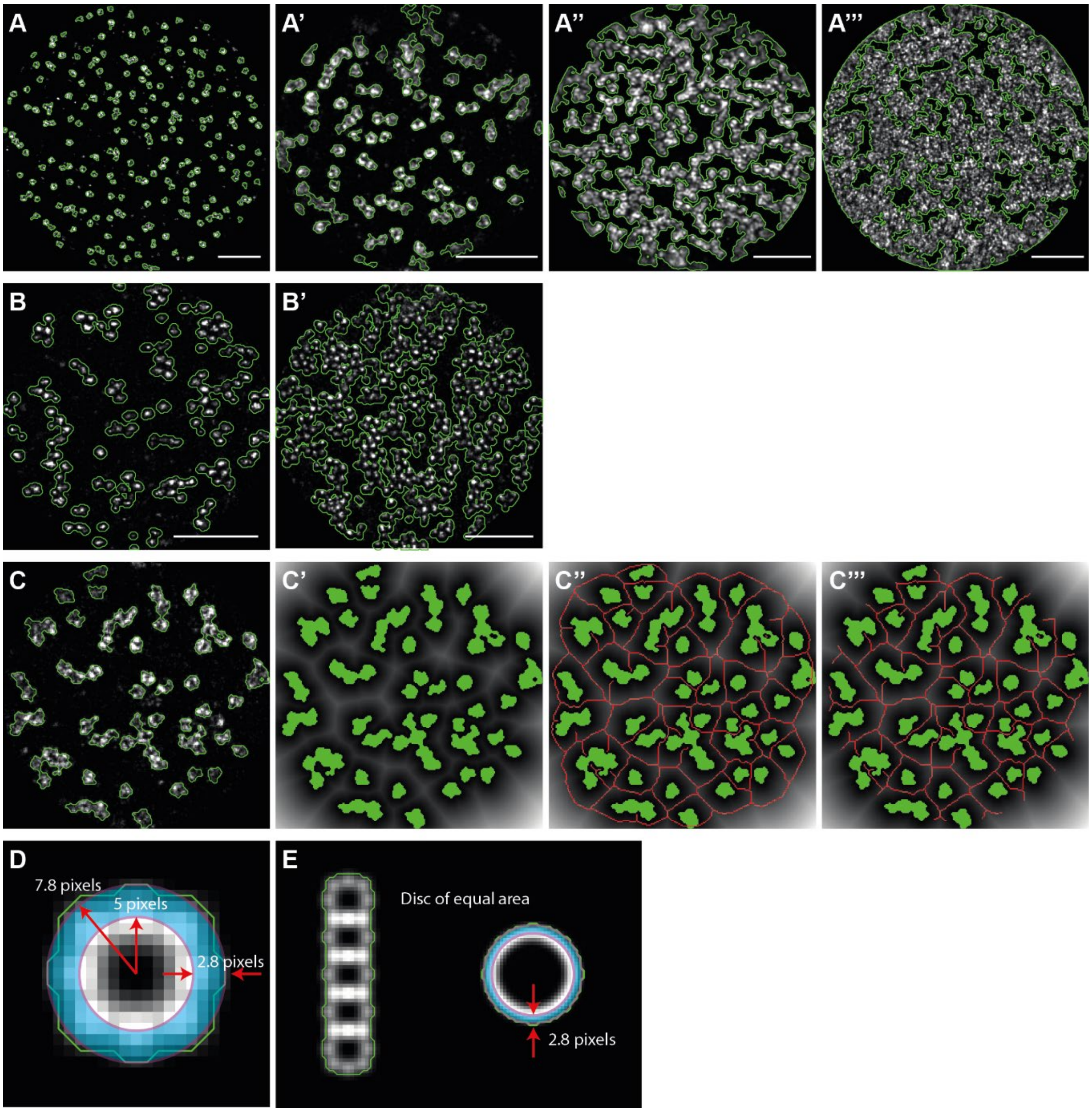

Fig. S8. Binarization and morphological image analysis. (A) A binarized super-resolved image of NPCs in the peripodial membrane of the *Drosophila* wing disc. The NPCs cover 5% of the nuclear membrane. **(A')** A binarized super-resolved image of NPCs in a follicle cell in a *Drosophila* stage 8 egg chamber ectopically expressing Lamin C. The NPCs cover 13% of the nuclear membrane. (**A''**) A binarized super-resolved image of NPCs in a nurse cell in a *Drosophila* stage 3 egg chamber with 55% NPC coverage of the nuclear membrane. (**A'''**) A binarized super-resolved image of NPCs in a nurse cell in a *Drosophila* stage 5 egg chamber with 75% NPC coverage of the nuclear membrane. **(B)** A binarized super-resolved image of NPCs labelled for Gle1 in a follicle cell in a *Drosophila* stage 8 egg chamber with 19% NPC coverage of the nuclear membrane. **(B')** A binarized super-resolved image of NPCs labelled for Gle1 in a nurse cell in a *Drosophila* stage 4 egg chamber with 53% NPC coverage of the nuclear membrane. Green lines indicate the boundaries between black and white pixels in binarized super-resolved images and are super-imposed on the original greyscale image for comparison purposes. **(C)** A binarized super-resolved image of NPCs in a follicle cell in a *Drosophila* stage 8 egg chamber. Green lines trace the boundaries between white and black pixels and are

Journal of Cell Science • Supplementary information

superimposed on the original grayscale image**. (C')** Euclidean distance transform of the binary image shown **in (C)**. The green areas indicate foreground pixels of the binary image in **(C)** and are superimposed on the grayscale distance transform image for convenience. The distance between the NPC clusters is estimated by the values of the distance transform image along the white ridges that run between the NPC clusters. **(C'')** A skeleton produced by morphologically thinning the binary image is superimposed as a red trace on the distance transformed image. **(C''')** The skeleton (red trace) after applying a round mask to exclude edge and peripheral pixels. The skeleton traces along the ridges in the distance transform image and provides a convenient way to select the pixels whose value corresponds to the distance between NPC clusters. **(D)** Simulated image of a single NPC. The green trace indicates the area occupied by a single NPC after the binarization process. This area is approximated by a disc with an effective radius of 7.8 pixels. To obtain the actual area occupied by the NPC ,the value of the effective radius is reduced by 2.8 pixels and the area of a disc with 5-pixel radius is used. **(E)** Simulated image of a linear arrangement of 5 NPCs. The green trace indicates the area occupied by this NPC cluster after binarization. To estimate the actual area, we first calculate the effective radius of a disk with equal area as the one produced by the binarization process of this linear NPC cluster. The effective radius of this disc is reduced by 2.8 pixels, and the area of a disc with this reduced radius is used to obtain an estimate on the actual area occupied by the linear NPC cluster. Scale bars for **(A, B)** are 1 µm.

Table S1. Halo Tag Effective Labelling Efficiency

Halo Ligand	Docking Strand	Oligo Storage Time	Fly Genotype	Total No. of NPCs	No. of Gle1 Positive	Percent of Gle1 Positive (%)	Effective Labelling Efficiency (%)
PBI-300-43	P1	>1.5 years	Gle1-Halo, heterozygous Nup160-SNAP, homozygous	210	142	67.62	26.29
PBI-300-43	P1	>1.5 years	Gle1-Halo, homozygous Nup160-SNAP, homozygous	724	611	84.39	20.72
O2	P3	< 1 month	Gle1-Halo, heterozygous Nup160-SNAP, homozygous	194	126	64.95	24.56
O2	P1	< 1 month	Gle1-Halo, heterozygous Nup160-SNAP, homozygous	674	578	85.76	43.24

Table S2. Guide Sites

	**Recognition sequence**
**Nup160**	
Guide1	ggccgttctgcagatgcgaa**cgg**
Guide2	gcagatgcgaacggatcttcagg
**Gle1**	
Guide 1	gtgcaatctgccgctcacgc**agg**
Guide 2	gccacaactttattgattga**agg**
**Nup188**	
Guide 1	ttcggttgtaaagtaattca**tgg**
Guide 2	cgactgagctgctagcccttc**gg**

Table S3. Imaging Conditions

Figure Number	Imager Stand and Concentration	Docking strand(s)	Slide Type	Oxygen Scavengers	Frame Rate (Hz)	Number of Frames	Laser Power (at the objective back aperture)
Fig. 2	P3-Cy3B 0.5 nM	Halo-P3	8-well chambered coverglass	PCA PCD Trolox	4	60,000	1.1 mW (546 nm)
Fig. 3A	Cy3B Lifeact 5 nM	--	Concavity slide	20 mM Sodium Sulfite	8	100,000	1.52 mW (546 nm)
Fig. 3D	Cy3B Lifeact 1 nM	--	Concavity slide	20 mM Sodium Sulfite	8	100,000	1.52 mW (546 nm)
Fig. 3G	Cy3B Lifeact 1 nM	--	8-well chambered coverglass	PCA PCD Trolox	8	60,000	0.87 mW (546 nm)
Fig. 4A-C	Cy3B Lifeact 1nM and P1-Atto643 1nM	Halo*-P1	8-well chambered coverglass	PCA PCD Trolox	4	60,000	0.87 mW (546 nm) and 2.47 mW (642 nm)
Fig. 4D-F	P2-Cy3B 1nM and P1 Atto643 0.5nM	Halo*-P1 and SNAP-P2	8-well chambered coverglass	PCA PCD Trolox	4	60,000	0.87 mW (546 nm) and 2.47 mW (642 nm)
Fig. 5A-H	P1-Atto643 0.2nM	Halo*-P1	8-well chambered coverglass	PCA PCD Trolox	4	60,000	2.47 mW (642 nm)
Fig. 5J	P1-Cy3B 2 nM	Halo*-P1	35 mm glass-bottomed dish	Oxidase, Catalase, Glucose	NA	25,000	Imaged on Nikon N-Storm system.
Fig. 6	P1-Atto643 0.5nM	Halo*-P1	8-well chambered coverglass	PCA PCD Trolox	4	60,000	2.47 mW (642 nm)
Fig. 7	P1-Atto643 0.2nM	Halo*-P1	8-well chambered coverglass	PCA PCD Trolox	4	60,000	2.47 mW (642 nm)
Fig. 8A	P1-Atto643 0.2 nM	Halo*-P1	8-well chambered coverglass	PCA PCD Trolox	4	60,000	2.47 mW (642 nm)
Fig. 8D-G	P1-Cy3B 0.8 nM	Halo-P1	8-well chambered coverglass	PCA PCD Trolox	4	60,000	0.86 mW (546 nm)
Fig. S10	P1- Atto643 0.2 nM	Halo*-P1	8-well chambered coverglass	PCA PCD Trolox	4	60,000	2.47 mW (642 nm)
Fig. S11C, D	P1 -Atto643 0.5 nM	Halo*-P1	8-well chambered coverglass	PCA PCD Trolox	4	60,000	1.1 mW (642 nm)

* denotes docking strands conjugated with PBI-300-43 halo ligand.
